# Integration of Pseudotargeted Metabolomics and Microbiomics Reveals That Hugan Tablets Ameliorate NASH with Liver Fibrosis in Mice by Modulating Bile Acid Metabolism via the Gut Microbiome

**DOI:** 10.3390/metabo15070433

**Published:** 2025-06-24

**Authors:** Wenran Dong, Ying Wang, Huajinzi Li, Huilin Ma, Yingxi Gong, Gan Luo, Xiaoyan Gao

**Affiliations:** School of Chinese Materia Medica, Beijing University of Chinese Medicine, Beijing 102488, China; 202153041@bucm.edu.cn (W.D.); wangy174@bucm.edu.cn (Y.W.); lhjz03211@gmail.com (H.L.); mahuilin0621@gmail.com (H.M.); gongyingxi1@gmail.com (Y.G.)

**Keywords:** Hugan tablets, nonalcoholic steatohepatitis, hepatic fibrosis, metabolomics, gut microbiome, bile acids

## Abstract

**Background/Objectives**: Non-alcoholic steatohepatitis (NASH) carries a high risk of developing hepatic fibrosis. Hugan tablets (HGTs), a traditional Chinese medicine, have exhibited potent anti-hepatic fibrosis effects, though the underlying mechanisms remain unclarified. This study aims to assess the efficacy of HGTs against NASH-related liver fibrosis in mice and investigate the underlying mechanisms via the integration of pseudotargeted metabolomics and microbiomics. **Methods**: C57BL/6 mice were fed a choline-deficient, ethionine-supplemented (CDE) diet and treated with HGTs. The therapeutic effects of HGTs in CDE mice were assessed. The underlying mechanism of HGTs was investigated by the integration of microbiomics, a pseudo-sterile model, untargeted followed by pseudotargeted metabolomics, and molecular docking. **Results**: HGTs alleviated NASH-related hepatic fibrosis in CDE mice and restored the composition of the gut microbiota. The depletion of the gut microbiota eliminated the anti-hepatic fibrosis effect of HGTs. HGTs increased intestinal 7-ketolithocholic acid and tauroursodeoxycholic acid via 7α/β-hydroxysteroid dehydrogenase (7α/βHSDH), while reducing deoxycholic acid (DCA) and taurodeoxycholic acid through inhibition of bile acid 7α-dehydratase (BaiE), leading to lower hepatic DCA. Six intestinal components of HGTs interacted with 7αHSDH, 7βHSDH, and BaiE, which are expressed in the bacterial genera altered by HGTs. **Conclusions**: HGTs alleviate NASH fibrosis by reshaping the gut microbiome, acting on microbial BA-metabolizing enzymes, and regulating the BA metabolism in the liver and gut.

## 1. Introduction

Non-alcoholic steatohepatitis (NASH) is an advanced form of non-alcoholic fatty liver disease, which is marked by hepatic steatosis, inflammation, hepatocyte injury and a variable degree of fibrosis [1,2]. In 2023, the prevalence of NASH among adult participants in China was 44.39%, with 20–30% of NASH patients progressing to fibrosis over 3 years [3,4]. The risk of hepatocellular carcinoma in NASH patients with advanced fibrosis is eight times higher than in patients with early liver fibrosis, and the risk of mortality is 6–13 times higher [5]. Accordingly, NASH presents a significant risk for the development of hepatic fibrosis, which is a pivotal factor in the advancement of the disease [6]. Hepatic fibrosis is widely recognized as a potentially reversible pathological process. Therefore, there is an urgent need to explore safe and effective treatment strategies to ameliorate NASH-related hepatic fibrosis.

The progression and exacerbation of hepatic fibrosis are regulated by gut microbiota metabolites such as bile acids (BAs), choline, trimethylamine oxide, short-chain fatty acids and so on. Among them, BAs are involved in a multitude of biological processes relevant to NASH fibrosis, including glucolipid metabolism, hepatic lipogenesis, cellular impairment, inflammation, and fibrosis [7,8]. Dysregulation of BA metabolism and perturbation of composition may contribute to exacerbated inflammation and increased fibrosis in the liver [9]. Several clinical cohorts have reported that circulating BA profiles in NASH patients at varying stages of hepatic fibrosis are altered significantly [10,11]. Several microbial genes encode enzymes that are involved in the biotransformation of BAs. For example, BaiE enzymes from *Eggerthella lenta* and *Clostridium scindens* catalyze the biotransformation of cholic acid (CA) to deoxycholic acid (DCA) [12,13]. Furthermore, two key enzymes for the bioconversion of CDCA to ursodeoxycholic acid7α-hydroxysteroid dehydrogenase (7αHSDH) and 7β-hydroxysteroid dehydrogenase (7βHSDH) have been identified in the gut microbiome [14]. BA metabolizing enzymes are implicated in BA dysbiosis in gastrointestinal disease [15,16]. Targeting BA-metabolizing enzymes and gut microbes encoding these enzymes may provide a potential strategy for the treatment of NASH-associated liver fibrosis.

Hugan tablets (HGTs) are Chinese medicine compound preparation that inhibit the occurrence of liver fibrosis and are used for the treatment of NASH [17,18,19]. HGTs are composed of six medicinal herbs: *Bupleurum chinense* DC., *Artemisia scoparia* Waldst. & Kit., *Schisandra chinensis* (Turcz.) Baill., *Isatis tinctoria* L., Pulvis Fellis Suis, and *Vigna radiata* (L.) R. Wilczek. The plant names were verified with MPNS (http://mpns.science.kew.org, accessed on 6 October 2024). Pharmacological studies indicated that HGTs mitigate gut microbiome dysbiosis [20]. In the early stages of this research, we observed that HGTs reduced the level of total bile acids (TBA) in the liver of NASH mice. However, whether HGTs ameliorate NASH fibrosis by targeting enzymes to modulate BAs metabolism remains unclear.

The choline-deficient ethionine-supplemented (CDE) diet can be utilized to establish a mouse model of NASH, with earlier studies demonstrating that 21-day feeding induces hepatic fibrosis in mice [21,22,23]. Therefore, we first used CDE diet-induced NASH mice to clarify the effects of HGTs in improving hepatic fibrosis. Then, we used 16S rRNA sequencing, untargeted metabolomics, and pseudotargeted metabolomics to identify potential key gut microbes and BAs that are significantly altered by HGTs. Based on the enzymatic pathways for BA biotransformation by gut bacteria, we identified three BA-metabolizing enzymes as candidate targets of HGTs. Ultimately, molecular docking analysis revealed that six intestinal ingredients target these three enzymes, thereby regulating microbial bile acid metabolism, restoring the hepatic and intestinal BA profile, alleviating hepatic fibrosis in mice. Our research preliminarily elucidated the interaction between TCM and BA-metabolizing enzymes in improving NASH, deepened the understanding of the antifibrotic mechanism of HGTs, and provided a new perspective for uncovering the therapeutic mechanisms of TCM.

## 2. Materials and Methods

### 2.1. Chemicals and Reagents

Hugan Tablets (batch No. Z20003336) were obtained from Sunflower Pharmaceutical Group Co., Ltd. (Harbin, China). A choline-deficient diet was purchased from SPF (Beijing) Biotechnology Co., Ltd. (Beijing, China). Mouse ELISA kits of ALT (KT2871-A), AST (KT30280-A), TC (KT30242-A), TG (KT30053-A), TBA (KT30100-A), VLDL (KT9186-A), HA (KT30890-A), Col IV (KT2297-A), PC III (KT 31017), LN (KT2161-A), 7αHSDH (KT0474-MA), 7βHSDH (KT0480-MA), and BaiE (KT0540-MA) were provided by Jiangsu Meimian Industrial Co., Ltd. (Yancheng, Jiangsu, China). Vancomycin(J16HY185103), metronidazole (J10GS153398), neomycin sulfate (J03HS183974), ampicillin (RH677914), ethionine (O15HS197946), and tiopronin (H29S9Z71288) were obtained from Shanghai Yuanye Bio-Technology Co., Ltd. (Shanghai, China). Pentobarbital (G1820091) was purchased from Shanghai Aladdin Biochemical Technology Co., Ltd. (Shanghai, China). Liquid chromatograph mass spectrometer-grade acetonitrile, formic acid, and methanol were obtained from Thermofisher Scientific (Waltham, MA, USA).

### 2.2. Animals

Forty-two C57BL/6 male mice (6 weeks old, SPF) were kept in the experimental animal center of Beijing University of Chinese Medicine under control conditions: 21–25 °C temperature, 55–65% humidity, and a 12 h/h light–dark cycle. The protocols of the animal experiment were approved by the Animal Care and Ethics Committee of Beijing University of Chinese Medicine (approval number: BUCM-4-2022081902-3035; approval date: 19 August 2022).

### 2.3. Experimental Design

#### 2.3.1. The Pharmacological Effects of HGTs on Hepatic Fibrosis in Mice with NASH

**Figure 1:**
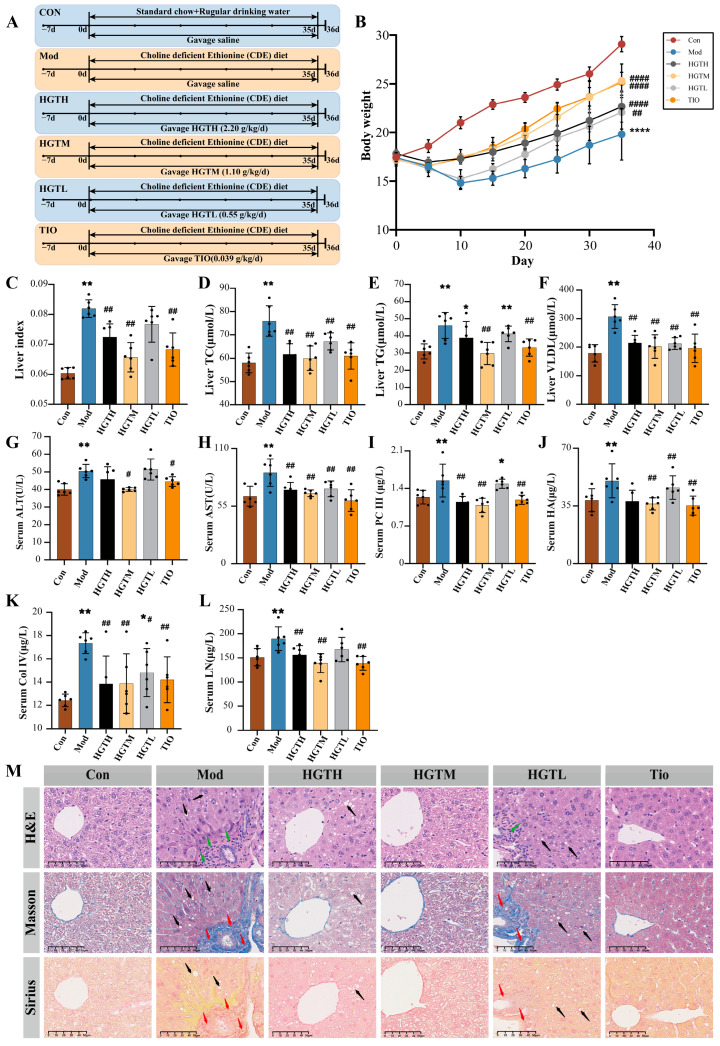
The effects of HGTs on hepatic fibrosis of NASH mice. (**A**) Flowchart of experimental design. (**B**) Body weight changes. (**C**) Liver index. (**D**) The level of TC in the liver. (**E**) The level of TG in the liver. (**F**) The level of VLDL in the liver. (**G**) The level of ALT in serum. (**H**) The level of AST in serum. (**I**) The level of PC III in serum. (**J**) The level of HA in serum. (**K**) The level of Col IV in serum. (**L**) The level of LN in serum. (**M**) Typical H&E, Masson, and Sirius staining sections (100×, scale bar length is 50 μm) of liver tissues from mice in each group. Black arrows mark the steatosis of hepatocytes. Red arrows mark the fibrosis area. Green arrows point to the inflammatory cell. The number of animals in each group is six. Compared with the Con group, * *p* < 0.05, ** *p* < 0.01, **** *p* < 0.0001; compared with the Mod group, ^#^ *p* < 0.05, ^##^ *p* < 0.01, ^####^ *p* < 0.0001.

After seven days of acclimatization, 36 mice were randomly divided into six groups (*n* = 6): control group (Con), model group (Mod), high-dose HGT group (HGTH), medium-dose HGT group (HGTM), low-dose HGT group (HGTL), and tiopronin group (TIO). The mice of the Con group were given free access to chow and sterile water, while the mice of other groups were given the choline-deficient diet, with 0.1% ethionine added to the water to establish hepatic fibrosis over a period of 35 days. At the same time, HGTH (2.20 g/kg), HGTM (1.10 g/kg), HGTL (0.55 g/kg), and TIO (tiopronin, 0.039 g/kg) mice were administered the respective drugs daily by gavage, while mice of the other two groups (Con and Mod) were delivered the same volume of saline by intragastric administration with. The schematic diagram of the experiment is illustrated in Figure 1A.

#### 2.3.2. Antibiotic Cocktail Animal Experiment

After seven days of acclimatization, 6 mice were used as a pseudo-sterile model group (HGTs-ABX). All these mice were applied to construct the NASH models in accordance with the above-mentioned experimental process. At the same time, an antibiotic mixture (ABX) cocktail (neomycin 100 μg/mL, ampicillin 50 μg/mL, vancomycin 0.5 mg/mL, and metronidazole 100 μg/mL) was dissolved in the drinking water, allowing the mice free access to eliminate the gut microbiome [24,25].

#### 2.3.3. General Observations and Sample Collection

Each mouse was weighed and recorded every two days. At the end of the experiment, mice were fasted for 12 h and anesthetized with 4% pentobarbital (1 mL/kg). Eyeballs were removed for blood collection. Livers were obtained, weighted to calculate the index scores, and stored at −80 °C. Colonic contents and feces were harvested and preserved at −80 °C.

### 2.4. Enzyme-Linked Immunosorbent Assay

The levels of alanine transaminase (ALT), aspartate transaminase (AST), total cholesterol (TC), total triglyceride (TG), TBA, very low-density lipoprotein (VLDL), collagen type IV (Col IV), hyaluronic acid (HA), laminin (LN), collagen type III N-peptide (PC III), 7αHSDH, 7βHSDH, and BaiE were quantified following the instructions provided with the ELISA kits.

### 2.5. Histopathological Analysis

Liver tissues were fixed in 4% paraformaldehyde, followed by dehydration, embedding, and sectioning into 4 μm paraffin sections. Staining was performed using hematoxylin and eosin (H&E), Masson’s trichrome (Masson), and Sirius red. Liver tissues were pathologically evaluated.

### 2.6. 16S rDNA Gene Sequencing Analysis of Mouse Colonic Contents Samples

Total microbial genomic DNA was extracted from colonic contents using the E.Z.N.A.^®^ Soil DNA Kit (Omega Bio-tek, Norcross, GA, USA) in accordance with manufacturer’s protocols. Amplification of the V3-V4 hypervariable region of the bacterial 16S rRNA gene was performed using primer pairs 338F (5′-ACTCCTACGGGAGGCAGCAG-3′) and 806R (5′-GGACTACHVGGGTWTCTAAT-3′) on a T100 Thermal Cycler PCR thermocycler (BIO-RAD, Hercules, CA, USA). The PCR product was purified and quantified with a Qubit 4.0 Fluorometer (Thermo Fisher Scientific, Waltham, MA, USA). Equimolar amounts of purified amplicons were pooled and subjected to paired-end sequencing on the Illumina Nextseq 2000 platform (Illumina, San Diego, CA, USA). The variation in the intestinal bacteria among three groups was evaluated by α-diversity including Ace, Chao, Simpson, and Shannon, as well as β-diversity including principal coordinate analysis (PCoA). Linear discriminant analysis (LDA) combined with effect size measurements (LefSe) was applied to determine bacteria exhibiting significant differences in abundance. Phylogenetic investigation of communities by reconstruction of unobserved states (PICRUSt2) was utilized to predict the functional pathways of the microbial flora. OTU encoded proteins were predicted by PICRUSt2 and annotated against the KEGG database to screen for OTUs that potentially encode BA-metabolizing enzymes.

### 2.7. Untargeted Metabolomics Assay

#### 2.7.1. Sample Preparation

For the liver samples, 800 μL of 70% (*v*/*v*) cold aqueous methanol was added to 100 mg of liver sample, homogenized, and then centrifuged at 4 °C and 13,000 rpm for 10 min. The supernatant was evaporated under nitrogen and redissolved in acetonitrile/water (*v*/*v*: 50/50) solvent before analysis. For the colonic contents sample, 400 μL of 70% (*v*/*v*) cold aqueous methanol was added to 40 mg of colonic contents, homogenized, and then centrifuged at 4 °C, 13,000 rpm for 10 min. The supernatant was evaporated under nitrogen and redissolved in acetonitrile/water (*v*/*v*: 50/50) solvent before analysis. To check on the stability of the system, quality control (QC) samples, which were mixed from 20 μL of each individual sample, were detected after every ten tested samples. Moreover, six QC samples were injected sequentially before sample testing.

#### 2.7.2. UPLC-Q-Exactive-MS

The UPLC-Q-Exactive-MS analysis was conducted using a Vanquish UPLC instrument coupled with a Q Exactive Orbitrap high-resolution mass spectrometer (Thermo Fisher Scientific, Waltham, MA, USA). The chromatographic analysis was carried out on an ACQUITY UPLC HSS T3 column (2.1 mm × 100 mm, 1.7 μm). The mobile phase was 0.1% formic acid water (A) and 0.1% formic acid acetonitrile (B). The gradient was established as follows: 0–0.5 min, 1% B; 0.5–4 min, 1–20% B; 4–8 min, 20–100% B; 8–9 min, 100% B; 9–9.5 min, 100–1% B; 9.5–12 min, 1% B, with a column temperature at 38 °C. The flow rate was set to 0.3 mL/min, and the injection volume was 3 μL.

Mass spectrometric data were equipped with an electrospray ionization source (ESI). Full scan/data-dependent MS2 (dd-MS2) in positive and negative ion modes was performed. The parameters were meticulously set as follows: spray voltage set at 3.5 kV or −2.8 kV; capillary temperature, 320 °C; auxiliary gas heater temperature, 350 °C; sheath gas, 40 arb; auxiliary gas, 10 arb. The resolutions of the MS and MS2 acquisitions were set at 350,000 FWHM and 17,500 FWHM. The scan range spanned from 80 *m*/*z* to 1000 *m*/*z*. The normalized collision energies (NCEs) applied were 15, 30, and 45 eV. The top 10 most intense ions were selected to perform MS2 acquisition.

#### 2.7.3. Data Analysis

Raw data were preprocessed using Xcalibur^TM^ 4.1 software (Thermo Fisher Scientific Inc., Waltham, MA, USA). Compound Discoverer 3.3 software (Thermo Fisher Scientific Inc., Waltham, MA, USA) was used for peak identification and peak extraction. Principal component analysis (PCA) was performed using Origin2024 software (Origin Lab, Northampton, MA, USA) and orthogonal partial least squares-discriminant analysis (OPLS-DA) was performed using Simca 14.1 software (Umetrics AB, Umea, Sweden). Metabolites with VIP (variable importance in the projection) > 1, *p* < 0.05, and FC > 2 or FC < 0.5 were selected as differential metabolites. Then, differential metabolites were identified based on the fragmentation information and accurate mass collected using MS data combined with the Human Metabolome Database (HMDB, https://hmdb.ca/, accessed on 8 October 2024). Next, metabolic pathway enrichment analysis of the differential metabolites was performed using Metaboanalyst 6.0 (https://www.metaboanalyst.ca/, accessed on 10 October 2024).

### 2.8. Pseudotargeted Metabolomics Assay

Pseudotargeted analysis was performed using the same liquid chromatograph (LC) conditions and UPLC system as described in Section 2.7.2. Mass detection was carried out in negative mode with a full scan at a mass resolution of 70,000 in the mass range of *m*/*z* 85–1200. Parallel reaction monitoring (PRM) was used to acquire pseudotargeted data. The remaining parameters were set the same as in the untargeted metabolomics analysis. The chemical formula, molecular mass of BAs, and collision energy (CE) parameters are shown in Table 1. Xcalibur 4.1 software was used to integrate the peak area of each compound measured in the PRM mode, Simca 14.1 software was used for OPLS-DA analysis, and SPSS 25.0 statistical software was used for one-way analysis of variance (ANOVA).

### 2.9. Literature Mining and Database Searching

Literature searches were conducted in Web of Science and PubMed utilizing keywords such as “gut microbiome + bile acids” and “gut microbiome + metabolic pathways + bile acids” to incorporate the most up-to-date research. Five databases including Virtual Metabolic Human database (VMH, https://www.vmh.life/, accessed on 15 October 2024), Bacterial diversity metadatabase (BacDive, https://bacdive.dsmz.de/, accessed on 17 October 2024), Microbiota-Active Substance Interactions database (MASI, http://www.aiddlab.com/MASI/, accessed on 17 October 2024), gutMEGA (http://gutmega.omicsbio.info, accessed on 17 October 2024), and gutMDisorder (http://bio-annotation.cn/gutMDisorder/home.dhtml, accessed on 19 October 2024) were utilized to identify the microbiota with the capacity to *metabolize BAs*.

### 2.10. Molecular Docking

Using Pubchem (https://pubchem.ncbi.nlm.nih.gov/, accessed on 6 November 2024), the SDF files of the ingredients were acquired, and their three-dimensional (3D) structures were optimized with Discovery Studio software (Dassault Systèmes, San Diego, CA, USA). The 3D crystal structures of 7αHSDH, 7βHSDH, and BaiE were obtained from the Research Collaboratory for Structural Bioinformatics (RCSB) Protein Data Bank (PDB) database (https://www.rcsb.org/, accessed on 6 November 2024) and preprocessed in Discovery Studio software by removing water molecules and ions. Molecular docking processes were subsequently conducted in Discovery Studio to evaluate the binding affinities between ligands and proteins. The docking results were further visualized using PyMOL 3.0 software (DeLano Scientific LLC, South San Francisco, CA, USA).

### 2.11. Statistical Analysis

Data are presented as the mean ± SD. SPSS 25.0 (IBM, Armonk, NY, USA) and GraphPad Prism 8.0 software (GraphPad Software, San Diego, CA, USA) were used for statistical analysis and visualization. The group comparisons were statistically analyzed using one-way ANOVA. Statistical significance was defined as *p* < 0.05.

## 3. Results

### 3.1. HGTs Alleviate NASH-Related Hepatic Fibrosis in CDE Mice

Compared with the Con group, mice of the Mod group showed a significant decrease in body weight, with slow weight gain subsequently, while the liver index increased significantly (*p* < 0.01). HGTH, HGTM, HGTL, and TIO mice exhibited varying degrees of weight recovery, accompanied by liver index reductions (Figure 1B,C). The biochemical indicators of hepatic lipids were used to evaluate the efficacies of HGTs. Relative to the Con mice, Mod mice showed higher levels of TC, TG, and VLDL, while HGTs and TIO suppressed the levels of these lipids (Figure 1D–F). ALT and AST are markers of liver function. LN, HA, Col IV, and PCIII are markers indicating the degree of hepatic fibrosis. Compared to the Con group, significantly elevated levels of ALT, AST, LN, HA, Col IV, and PC III were observed in the mice of the Mod group. After treatment with HGTs or TIO, the levels of these indicators decreased to varying degrees. Differences among three dose groups of HGTs were not statistically significant. However, all these indicators were statistically lower in the HGTM in comparison with the Mod group (Figure 1G–L). Furthermore, H&E revealed lipid droplet accumulation in hepatocytes and inflammatory cell infiltration around vessels in Mod mice, while these pathological features were reversed in HGTM and TIO. Compared with the Con group, Masson staining showed fiber proliferation in the Mod group, while the HGTM and TIO showed reduced fiber proliferation (Figure 1M). In summary, HGTs exhibit anti-hepatic fibrosis effects in the mouse model, and the alleviation effect of liver fibrosis was observed most significantly in the HGTM. Therefore, samples of the HGTM were selected as the HGT group for further studies.

### 3.2. Effects of HGTs on the Intestinal Flora of Mice with NASH Fibrosis

As the number of OTUs increased, the Pan-Core curves tended to flatten, indicating that sequence depth was adequate (Supplementary Figure S1A,B). The rank abundance curve indicated a uniform distribution of species (Supplementary Figure S1C). Accordingly, all samples met the requirements, and the sequencing data, which is reasonable, can be used for subsequent analysis.

#### 3.2.1. Diversity Analysis

**Figure 2:**
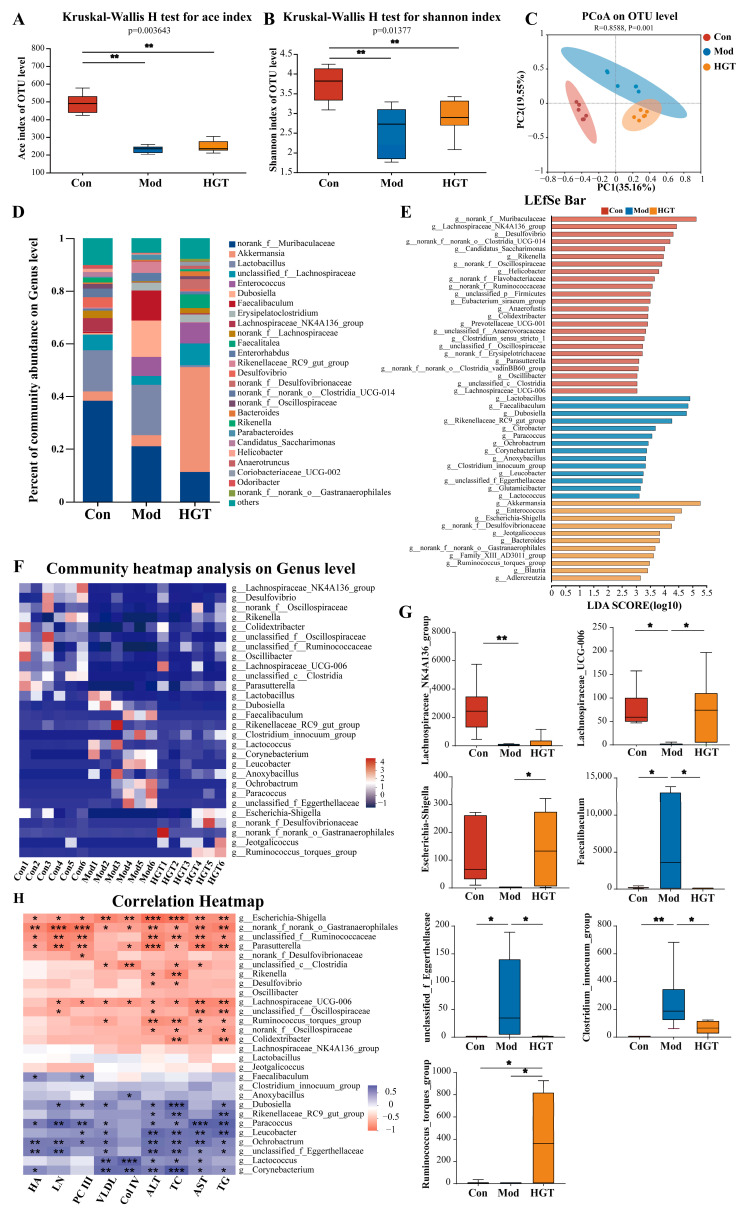
HGTs modulated the structure and function of the gut microbiota in mice with NASH fibrosis. (**A**) Ace indexes. (**B**) Shannon indexes. (**C**) PCoA at the OTU level. (**D**) The compositions of intestinal bacteria in each group at the genus level. (**E**) Histogram of the LDA values of dominant genera based on Lefse analysis. (**F**) Heatmap of potential key genera in the HGT treatment. (**G**) The relative abundances of the *Lachnospiraceae NK4A136 group*, *Lachnospiraceae UCG 006*, *Escherichia-Shigella*, the *Ruminococcus_torques_group*, *Faecalibaculum*, *the Clostridium innocuum group*, *and unclassified_f_Eggerthellaceae*. (**H**) Pearson’s correlation analysis between the abundance of 28 potential key genera and the levels of TC, VLDL, Col IV, HA, LC, PC III, TG, ALT, and AST. The number of animals in each group is six, * *p* < 0.05, ** *p* < 0.01, *** *p* < 0.001.

Ace, Shannon, and Chao indices in the Mod group were lower than those in the Con and HGT groups (Figure 2A,B, Supplementary Figure S1D). There was no obvious difference in the Simpson index among the three groups (Supplementary Figure S1E). PCoA was performed to compare the compositional similarity of the intestinal community among three groups. As shown in Figure 2C, the PCoA graph evaluating the differences in the composition of OTU data in each group demonstrated significant separation among the three groups.

#### 3.2.2. Structural Composition Analysis

Alterations in gut microbiota structure are predictive of functional changes. Therefore, this part focused on the structural changes in gut microbiota at the phylum and genus levels. As presented in Supplementary Figure S1F, at the phylum levels, *Firmicutes* and *Bacteroidetes* are the two major communities in gut microbiota. Compared to mice in the Con group, the relative abundance of *Firmicutes* was elevated in mice of the Mod group, which was reversed after treatment with HGTs. At the genus level, the Mod group showed an increase in the proportions of *Lactobacillus, Enterococcus*, and *Faecalibaculum* compared to the Con group, while HGTs downregulated the proportions of *Lactobacillus* and *Faecalibaculum* (Figure 2D). In summary, HGTs mended the structural perturbations of the gut microbiome in mice with NASH fibrosis.

#### 3.2.3. Linear Discriminant Analysis Effect Quantity (LEfSe) and Differential Bacteria Genus Analysis

LefSe analysis was conducted to identify the dominant intestinal flora from phylum to genus (Supplementary Figure S1H). According to LDA (LDA score > 3) of gut bacterial genera, the characteristic genera of the Mod group included *Faecalibaculum*, *Paracoccus*, *and the Clostridium innocuum group*, while the predominant genera of the HGT group included *Akkermansia*, *Bacteroides*, *Escherichia-Shigella*, *and the Ruminococcus_torques_group* (Figure 2E). Subsequently, ANOVA analysis revealed that the abundance of 59 genera was significantly reverted by HGTs (*p* < 0.05). By combining Lefse analysis, we finally screened out 28 genera as potential key microbial genera in HGT treatment (Figure 2F). Among them, compared with the Mod group, HGTs increased the abundance of the *Lachnospiraceae NK4A136 group* and *Lachnospiraceae UCG 006*, which are specific genera of the Con group, also enriched *Escherichia-Shigella* and the *Ruminococcus_torques*_*group*. Additionally, HGTs suppressed the proliferation of characteristic genera in Mod mice, such as *Faecalibaculum*, *the Clostridium innocuum group*, and *unclassified_f_Eggerthellaceae* (Figure 2G).

#### 3.2.4. PICRUSt Functional Prediction

In this study, PICRUSt was used to predict the alterations in gut microbiome function in response to HGTs. Compared to the mice in the Con group, the Mod group exhibited 20 differential functional pathways (Supplementary Figure S1I). A total of 10 out of 20 pathways were reversed by HGTs, including steroid degradation, the TCA cycle, and other pathways (Supplementary Figure S1J). Most of these pathways were classified as being related to metabolism at the first level of the KEGG pathways.

#### 3.2.5. Correlation Analysis Between Gut Microbiota and Hepatic Fibrosis

Correlation analysis was performed between 28 potential key genera and biochemical indexes to further screen the genera that are highly associated with the antifibrotic effect of HGTs. The genera inhibited by HGTs, such as *Faecalibaculum*, *Ochrobactrum*, and *unclassified_f_Eggerthellaceae* were positively linked with most indicators. Conversely, the genera elevated by HGTs, such as *Escherichia-Shigella* and *Parasutterella* were negatively correlated with multiple indicators. These bacteria may serve as potential key genera for HGT treatment of hepatic fibrosis (Figure 2H).

### 3.3. ABX Attenuated the Anti-Hepatic Fibrosis Effect of HGTs in NASH Mice

**Figure 3:**
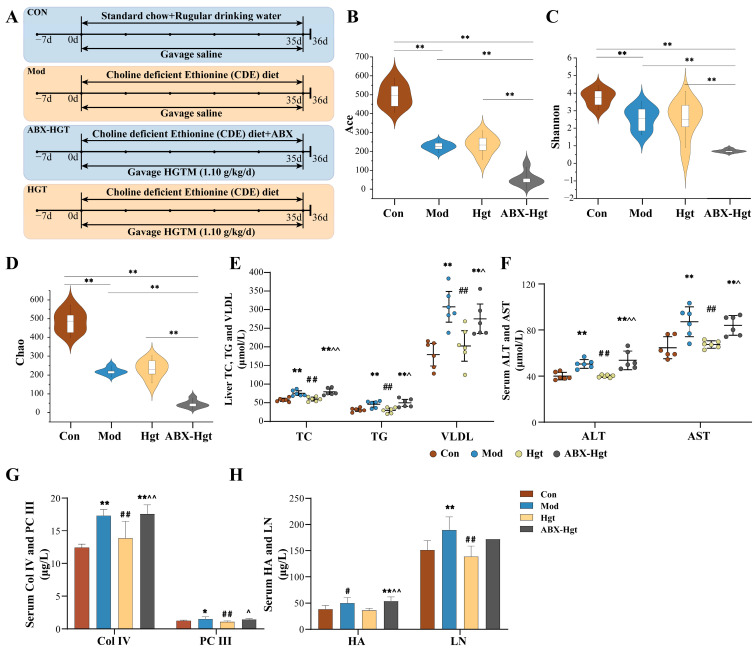

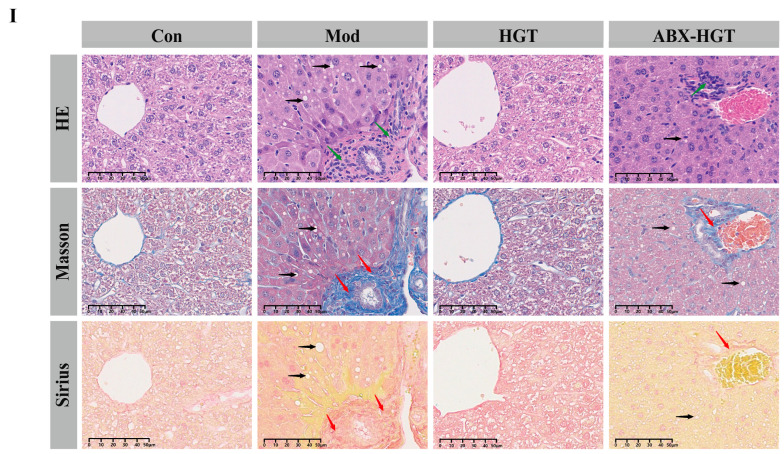
Effect of antibiotic cocktail on the gut microbiota and anti-hepatic fibrosis efficacy of HGTs. (**A**) Schedule flow chart of antibiotic cocktail animal experiment. Shannon (**B**), Ace (**C**), and Chao (**D**) indexes of alpha-diversity analysis. (**E**) Levels of TC, TG, and VLDL in the liver. (**F**) Levels of ALT and AST in the serum. (**G**) Levels of Col IV and PC III in the serum. (**H**) Levels of HA and LN in the serum. (**I**) Typical H&E, Masson, and Sirius staining sections (100×, scale bar length is 50 μm) of liver tissues. Black arrows point to the steatosis of hepatocytes. Red arrows point to the fibrosis area. Green arrows point to the inflammatory cell. The number of animals in each group is six. Compared with the Con group, * *p* < 0.05, ** *p* < 0.01; compared with the Mod group, **^#^** *p* < 0.05, **^##^** *p* < 0.01; compared with the HGT group, ^^^ *p* < 0.05, ^^^^ *p* < 0.01.

To determine whether intestinal flora mediates the anti-hepatic fibrosis effect of HGTs, we evaluated the therapeutic effects of HGTs following gut microbiota depletion with an ABX cocktail (Figure 3A). The concentration of DNA in the colonic contents of the mice in the ABX-HGT group was significantly lower than that in the HGT and other groups. Compared with the Con and Mod groups, Ace, Shannon, and Chao indices, as well as the number of OTUs, were significantly decreased in the ABX-HGT group. These results revealed that ABX-HGT mice had a significantly reduced gut bacterial community (Figure 3B–D, Supplementary Figure S3A–C). Reductions in liver index, TC, TG, AST, ALT, LN, HA, Col IV, and PC III observed in the HGT group were eliminated after ABX treatment (Figure 3E–H). Furthermore, lipid droplets and inflammatory cells were prominently observed in the livers of the HGTs-ABX group (Figure 3I). As we anticipated, ABX partially abolished the anti-hepatic fibrosis effect of HGTs in NASH mice, implicating that gut microbiota are involved in mediating the anti-hepatic fibrosis effect of HGTs. 

### 3.4. HGTs Regulate the Bile Acid Metabolism in the Liver and Colonic Contents of Mice with NASH Fibrosis

Generally, the gut microbiota regulates host metabolism mediated by their metabolites [26,27]. Thus, we speculated that HGTs ameliorate hepatic fibrosis of NASH mice via restoring metabolic homeostasis through small molecule messenger metabolites. To further identify metabolites affected byHGTs, untargeted metabolomics on the colonic contents and livers of mice were performed.

#### 3.4.1. Untargeted Metabolomics

##### Metabolic Profiles of Colonic Contents

**Figure 4:**
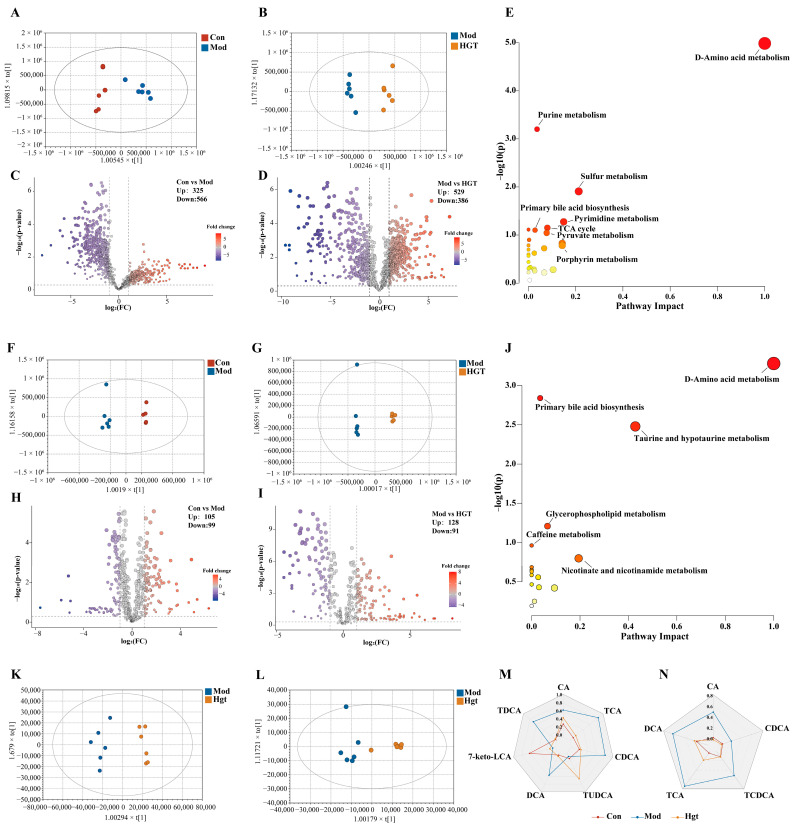
HGTs altered colonic contents and liver metabolism in the fibrosis of NASH mice. (**A**) OPLS-DA plots of colonic contents between Con and Mod. (**B**) OPLS-DA plots of colonic content between Mod and HGTs. (**C**) Volcano plot of colonic contents between Con and Mod. (**D**) Volcano plot of colonic contents between Mod and HGTs. (**E**) The KEGG pathway enrichment analysis of colonic content metabolites restored by HGTs. (**F**) OPLS-DA plots of livers between Con and Mod. (**G**) OPLS-DA plots of livers between Mod and HGTs. (**H**) Volcano Plot of livers between Con and Mod. (**I**) Volcano Plot of livers between Mod and HGTs. (**J**) KEGG pathway enrichment analysis of liver metabolites restored by HGTs. (**K**) OPLS-DA score plot of Mod vs. HGTs (colonic contents). (**L**) OPLS-DA score plot of Mod vs. HGTs (livers). Radar Plot for BAs in colonic contents (**M**) and livers (**N**) with significant differences in the Con group, Mod group, and HGT group. The abundance of each BA is normalized using min-max normalization. The number of animals in each group is six.

As shown in the PCA score plots (Supplementary Figure S2A), a trend of aggregation within each group, along with distinct separation between the three groups, was visually illustrated. OPLS-DA of positive and negative ion modes was constructed to clarify prospect differential metabolites (Figure 4A,B). Subsequently, the OPLS-DA model was validated based on the permutation tests (*n* = 200), and the results (*R*^2^ = 0.815, *Q*^2^ = 0.686) indicated that the model was effective and reliable (Supplementary Figure S2B,C). According to the criteria of VIP > 1, *p* < 0.05, and FC > 2 or < 0.5, the differential metabolites between Con and Mod and between Mod and HGTs were intersected (Figure 4C,D). Additionally, if the abundance of metabolites could be restored by HGTs, this metabolite was listed as a candidate. Next, all candidates were identified by searching the HMDB. Based on the above screening criteria, a total of 148 differential metabolites were obtained (Supplementary Table S1). In accordance with the HMDB and KEGG databases, these metabolites were predominantly classified into amino acids, lipids, and BAs (Supplementary Figure S2D). Finally, pathway enrichment analysis was performed using Metaboanalyst. Figure 4E shows the pathways regulated by HGTs, including D-amino acid metabolism, purine metabolism, and primary bile acid biosynthesis (Supplementary Table S3).

##### Liver Metabolic Profiles

The PCA chart showed obvious separation between Con and Mod, as well as between Con and HGTs (Supplementary Figure S2E). We constructed the OPLS-DA model to identify the differential metabolites (Figure 4F,G). The permutations of the OPLS-DA indicated that these models were reliable and could be used for further analysis (Supplementary Figure S2F,G). Differential metabolites were screened with VIP > 1, *p* < 0.05, and FC > 2 or < 0.5, resulting in 39 liver differential metabolites that were restored by HGTs, serving as potential key metabolites (Figure 4H,I, Supplementary Table S2). These metabolites were classified, and the highest proportions belonged to organic acids, lipids, and BAs (Supplementary Figure S2H). The 39 differential metabolites were loaded into Metaboanalyst for pathway enrichment analysis, revealing that HGTs restored multiple pathways perturbed in the Mod group, including D-amino acid metabolism, primary bile acid biosynthesis, and taurine and hypotaurine metabolism (Figure 4J, Supplementary Table S4). 

Clinical studies and basic research indicate that dysregulation of BA homeostasis is involved in the development and progression of NAFL, NASH, fibrosis, and cirrhosis [28,29]. In this research, we found that primary bile acid biosynthesis was a common differential metabolic pathway of colonic contents and liver regulated by HGTs. Thus, we further focus on the regulatory effect of HGTs on BAs in the colonic contents and liver, aiming to clarify the potential key metabolites associated with the anti-fibrosis effects of HGTs on NASH mice.

#### 3.4.2. Pseudotargeted Metabolomics

##### Fecal TBA Levels and Colonic Content BA Composition

We initially assessed alterations in total bile acid (TBA) within feces. In comparison with mice in the Con group, the TBA level was reduced in mice of the Mod group but was recovered after HGT treatment, underscoring the regulatory effects of HGTs on BAs (Supplementary Figure S4B). The pseudotargeted analysis was employed to investigate the BA profile in the colonic contents of three groups. The pseudotargeted transition overlap chromatogram was obtained on the PRM of UPLC-Q-Exactive-MS (Supplementary Figure S4D). The OPLS-DA score plots (Figure 4K) showed a clear separation between different groups (*R*^2^ = 0.954, *Q*^2^ = 0.894, Supplementary Figure S4F). BAs with significant intergroup differences (VIP > 1.0, *p* < 0.05) were identified, and those showing substantial correction following HGT intervention were selected as potential key BAs. Our findings demonstrated that, compared with the Mod group, HGTs markedly increased the abundance of primary BAs, including 7-keto LCA and TUDCA, while decreasing that of CA, CDCA, and TCA. Additionally, HGTs decreased the abundance of secondary BAs, including DCA and TDCA (Figure 4M).

##### Hepatic TBA Levels and BAs Composition

The results of liver TBA suggested that HGTs lowered the abnormally elevated TBA level in the Mod group (Supplementary Figure S4C). The overlap chromatogram was obtained on the PRM of UPLC-Q-Exactive-MS (Supplementary Figure S4E). Consistent with results from colonic contents, the data presented in Figure 4L revealed a tendency for aggregation within groups and separation between groups (*R*^2^ = 0.873, *Q*^2^ = 0.811, Supplementary Figure S4G). Under the criteria of VIP > 1 and *p* < 0.05, the relative abundance of individual BAs was significantly increased in the mice of the Mod group, while HGTs downregulated the abundance of primary BAs (CA, CDCA, TCA, and TCDCA) and secondary BAs (DCA) compared with the Mod group (Figure 4N). Therefore, these BAs may be potential key BAs for HGTs’s therapeutic effects on NASH fibrosis in mice (Table 2).

### 3.5. The Multidimensional Mechanism of HGTs Targeting BA-Metabolizing Enzymes to Regulate BA Profiles via Gut Microbiome in Improving Hepatic Fibrosis

#### 3.5.1. Correlation Analysis Between BAs and Hepatic Fibrosis

**Figure 5:**
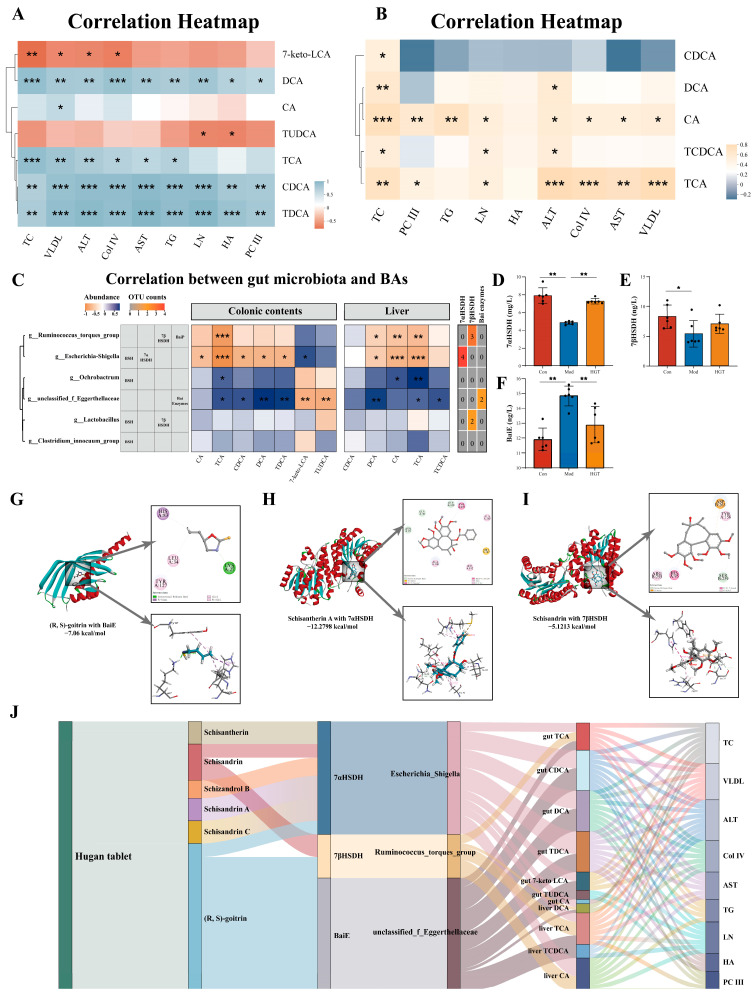
The potential mechanisms for HGTs to alleviate fibrosis by regulating BA profiles through the gut microbiome. (**A**) Correlation between markedly altered BAs in colonic contents and hepatic fibrosis indicators. (**B**) Correlation between markedly altered BAs in the liver and hepatic fibrosis indicators. (**C**) Correlation analysis between markedly altered intestinal and hepatic BAs and potential key genera with BAs-metabolizing capabilities. The level of 7αHSDH (**D**), 7βHSDH (**E**), and BaiE (**F**) in colonic contents. Molecular docking diagrams of (R, S)-goitrin to BaiE (**G**), schisantherin A to 7αHSDH (**H**), and schisandrin to 7βHSDH (**I**). (**J**) Interrelationship between HGTs, ingredients, BA enzymes, gut microbiota, BAs, and phenotypes of fibrosis. The number of animals in each group is six, * *p* < 0.05, ** *p* < 0.01, *** *p* < 0.001.

Within the heatmap (Figure 5A), intestinal CDCA, TDCA, DCA, and TCA were positively correlated with multiple hepatic fibrosis indicators, whereas 7-keto LCA negatively correlated with ALT, TC, VLDL, and Col IV, and TUDCA exhibited negative correlations with LN and HA. Hepatic CA, TCA, DCA, and TCDCA were positively correlated with several indexes (except HA), indicating the therapeutic effects of HGTs are associated with these BAs (Figure 5B).

#### 3.5.2. Identification of Potential Targets of HGTs in Regulating Bile Acid Metabolism

To clarify the relationship between the microbiome and BAs, the abundance of markedly altered BAs in the colonic contents and liver was related to the 28 potential key genera identified before. A total of 26 were associated with colonic content BAs reversed by HGTs (Supplementary Figure S5A), while 23 were linked to hepatic BAs reversed by HGTs (Supplementary Figure S5B).

To further identify which of the 28 potential key genera possess BA-metabolizing capabilities and may be involved in the observed alterations in intestinal and hepatic BAs, we first screened taxa with BA biotransformation potential through literature and databases. Among the genera correlated with BAs, six possess the capacity to metabolize BAs [8,15]. Then, the capacity of OTUs belonging to six differential genera to encode BA-metabolizing enzymes was predicted using PICRUSt. Among the genera enriched in the Con and HGT groups, four OTUs from *Escherichia-Shigella* had the potential to encode 7αHSDH (EC: 1.1.1.159), which catalyzes the biotransformation of CDCA to 7-keto LCA, while three OTUs from the *Ruminococcus_torques_group* encoded 7βHSDH, which facilitated the conversion of 7-keto LCA to UDCA. Two OTUs assigned to *unclassified_f_Eggerthellaceae*, enriched in the Mod group, were capable of encoding bile acid 7α-dehydratase (BaiE, EC: 4.2.1.106), which is involved in the bioconversion of CA to DCA (Figure 5C). The levels of the three enzymes in colonic contents changed accordingly. Compared with the Con group, the levels of 7αHSDH and 7βHSDH were lessened, while the level of BaiE was increased in the Mod group, and HGTs restored these alterations (Figure 5D–F).

In conclusion, the Mod group enabled the accumulation of intestinal genera encoding BaiE, which is involved in the biotransformation of CA to DCA. HGTs elevated the abundance of genera with the potential to encode 7αHSDH and 7βHSDH, which catalyze the conversion of CDCA to UDCA. These three BA-metabolizing enzymes may serve as potential targets of HGTs in regulating BA profiles.

#### 3.5.3. Molecular Docking of Bile-Acid-Metabolizing Enzymes and Active Ingredients of HGTs

As the intestinal components of HGTs were identified in our previous study, they were considered as candidate active ingredients for molecular docking with three enzymes [18]. The molecular docking results showed that six ingredients of HGTs exhibited high affinity for 7αHSDH, 7βHSDH and BaiE. The conformations with the highest binding scores included schisantherin A ~ 7αHSDH (−12.28 kcal/mol), (R, S)-goitrin ~ BaiE (−7.06 kcal/mol), and schisandrin ~7βHSDH (−5.12 kcal/mol) (Figure 5G–I). The results suggested that multiple active ingredients of HGTs may be attributed to interactions with these bile-acid-metabolizing enzymes. Full details of the calculation and corresponding results are presented in Supplementary Table S5.

#### 3.5.4. Multidimensional TCM-Enzyme-Intestinal Genera-Bile Acids-Disease Interaction

Based on the above data, the multidimensional relationships among six ingredients of HGTs, three BA-metabolizing enzymes, three differential genera, twelve regulated BAs, and nine phenotypes were constructed. In summary, HGTs reduced the abundance of *unclassified_f_Eggerthellaceae* while increasing that of the *Ruminococcus_torques_group* and *Escherichia-Shigella*, which are the three genera expressing BaiE, 7βHSDH, and 7αHSDH. A total of six ingredients of HGTs interacted with these enzymes, resulting in alterations in intestinal and hepatic BA profiles (Figure 5J). These findings suggest a potential mechanism by which HGTs exert antifibrotic effects on NASH mice. 

## 4. Discussion

Currently, there is confirmed evidence that HGTs have hepatoprotective activities [30] and anti-hepatic fibrosis effects [20], but the specific mechanism remains unclear. This study established a NASH-related fibrosis mouse model using a CDE diet, evaluated the efficacy of HGTs in the anti-hepatic fibrosis of NASH mice, observed the regulatory effect of HGTs on the gut microbiota and metabolites, and revealed the potential mechanism by which HGTs improve NASH with liver fibrosis in CDE mice.

In this study, weight loss, increased liver index, and elevated hepatic lipids were observed in the Mod group, while HGTs restored these indices to normal, indicating that HGTs could decrease liver lipids and cholesterol. PC III, Col IV, HA, and LN levels are important indicators for measuring liver inflammation activity and fibrosis [31]. HGTs reduced liver transaminase levels and contents of PC III, Col IV, and HA in NASH fibrosis mice, which demonstrated effects of HGTs on liver protection and anti-fibrosis.

Dysbiosis of the gut microbiota is one of the mechanisms in the pathogenesis of NASH [32]. Drugs targeting the gut microbiota may be a potentially effective treatment for NASH. Oral drugs can manipulate microbial composition, and this interaction is highly prevalent [33]. Therefore, we speculated that HGTs, as an orally administered drug absorbed through the gastrointestinal tract, may have an impact on the composition of the gut microbiota, which might be a potential target. Animal studies have shown that intestinal microbiome can increase intrahepatic fat, and the reduction in gut microbiome diversity is associated with NASH [34,35]. Our results showed hepatic lipid accumulation and reduction in gut microbiota diversity in Mod mice. However, HGTs increased the diversity of gut microbiota and correspondingly reduced liver TC and TG. It was found that NASH patients exhibited increased levels of *Firmicutes* [36]. A longitudinal study demonstrated a correlation between reduced intrahepatic triglycerides and a decline in the abundance of *Firmicutes* [37]. For CDE-fed mice, our data showed an increase in the abundance of *Firmicutes*, but this gut microbiota structure was restored by HGTs. At the genus level, the abundance of *Oscillibacter* was reduced in CDE-fed mice, but HGTs restored its relative abundance. In recent reports, *Oscillibacter* has been shown to effectively absorb and break down cholesterol, and individuals with a higher abundance of *Oscillibacter* in their gut typically exhibit lower cholesterol levels [38]. The conversion of cholesterol into bile acids in the liver represents a major fraction of cholesterol excretion [39]. Moreover, according to databases, several genera significantly altered by HGTs, including the *Ruminococcus_torques_group*, the *Clostridium innocuum group*, *Lactobacillus*, *Bacteroides*, and *Ochrobactrum*, are involved in BA metabolism. The results of untargeted metabolomics suggested that bile acids are the metabolite category significantly regulated by HGTs, with the primary bile acid synthesis pathway being one of the major metabolic pathways involved in both the intestine and liver. Therefore, we further focused on the effect of HGTs on hepatic and intestinal BAs metabolism.

The pathogenesis of NASH is associated with impaired hepatobiliary excretory function and functional micro-cholestasis [40,41,42]. Prolonged exposure of the liver to high concentrations of BAs leads to hepatocellular inflammation due to their toxicity, which subsequently results in liver fibrosis [43,44]. In our work, the TBA levels in the liver and serum of mice with NASH fibrosis were elevated, while TBA levels in feces were reduced, indicating the occurrence of intrahepatic cholestasis. HGTs reversed this trend by lowering hepatic and serum TBA while increasing fecal TBA, indicating enhanced BA excretion. As a nuclear bile acid receptor farnesoid X receptor agonist, UDCA enhances the hepatic excretion of BAs, thereby mitigating the accumulation of hepatotoxic BAs [45]. HGTs increased the abundance of its precursor 7-keto-LCA and its conjugate TUDCA in the intestine, potential promoting BA excretion. The levels of 7-keto-LCA and TUDCA were negatively correlated with Col IV, LN, and HA, suggesting that HGTs may alleviate liver fibrosis by promoting bile acid excretion through increasing 7-keto-LCA and TUDCA. DCA can activate hepatic stellate cells, thus accelerating the process of fibrosis [4,46]. Studies have shown that intestinal DCA level is significantly elevated in NAFLD patients, with this increase positively correlating with hepatic fibrosis progression [10]. In clinical trials, Pegbelfermin (PGBF), which was under development for the treatment of NASH, reduced the serum concentrations of DCA and its conjugates in patients [47,48]. Interestingly, HGTs effectively reduced the hepatic and intestinal levels of DCA and TDCA, which were positively correlated with fibrosis indicators. In summary, HGTs may relieve liver BAs stasis and reduce the abundance of DCA and its conjugates in both the liver and intestine, thereby alleviating NASH fibrosis in mice.

BAs are synthesized in the liver and secreted into the small intestine after a meal. Approximately 95% of BAs are resorbed and transported back to the liver for recycling once they reach the terminal ileum, the remaining 5% reach the colon, where they undergo biotransformation through specific enzymatic reactions, to generate secondary BAs. A portion of these secondary BAs can be transported to the liver via the portal vein, while the rest are excreted in the feces [49]. In the microbial biotransformaion of BAs, DCA is formed from CA through 7-dehydroxylation catalyzed by enzymes encoded by the polycistronic *bai*. At the same time, UDCA is generated from CDCA through epimerization catalyzed by 7αHSDH and 7βHSDH [8]. Our research indicated that the alterations in hepatic and intestinal BAs were associated with gut microbiome. The bai gene cluster for the multistep 7α/β-dehydroxylation pathway has been reported in *Eggerthella* spp. [15]. In this study, *unclassified_f_Eggerthellaceae* was positively correlated with intestinal DCA and TDCA. PICRUSt suggested that *unclassified_f_Eggerthellaceae* potentially encodes BaiE, while HGTs reduced its abundance. Genes encoding 7αHSDH and 7βHSDH have been cloned from *Escherichia coli* and the *Ruminococcus_torques_group*, respectively [50,51]. Correlation analysis indicated that *Escherichia-Shigella* was positively correlated with 7-keto-LCA. PICRUSt further suggested that these two genera potentially express 7αHSDH and 7βHSDH, and HGTs enriched these two genera. Given that the predictions by PICRUSt2 cannot directly reflect the expression of functional genes, we measured the level of the three enzymes in colonic contents. HGTs increased the levels of 7αHSDH and 7βHSDH while decreasing the level of BaiE in the colonic contents of mice. Six ingredients of HGTs ((R, S)-goitrin, schisandrin A, schisandrin C, schisandrin, schisantherin A, schizandrol B) exhibited high binding affinity for BaiE, 7αHSDH, and 7βHSDH. Indeed, the intestinal concentration of these ingredients has not been reported. Considering that traditional Chinese medicine formulas are absorbed through the digestive tract and subsequently enter the bloodstream, the prototypical metabolites of HGTs identified to be absorbed into the blood were selected for molecular docking. Therefore, we speculated that the anti-fibrotic effects of HGT might be attributed to the conversion of intestinal and hepatic BAs by three specific bacterial genera and that three enzymes may serve as candidate targets of HGTs.

To confirm that the gut microbiota is indispensable in the anti-fibrosis effect of HGTs, an ABX experiment was performed. Alterations in biodiversity and composition of intestinal bacteria were observed in HGT mice after antibiotic administration. Elevated serum levels of ALT, AST, HA, LN, Col IV, and PC III, along with increased inflammatory cell infiltration and fibrotic deposition in the liver, indicate ABX could weaken the anti-liver fibrosis effect of HGTs to a certain extent. It could be confirmed that gut microbiota is indispensable for the anti-fibrosis effect of HGTs.

## 5. Conclusions

**Figure 6:**
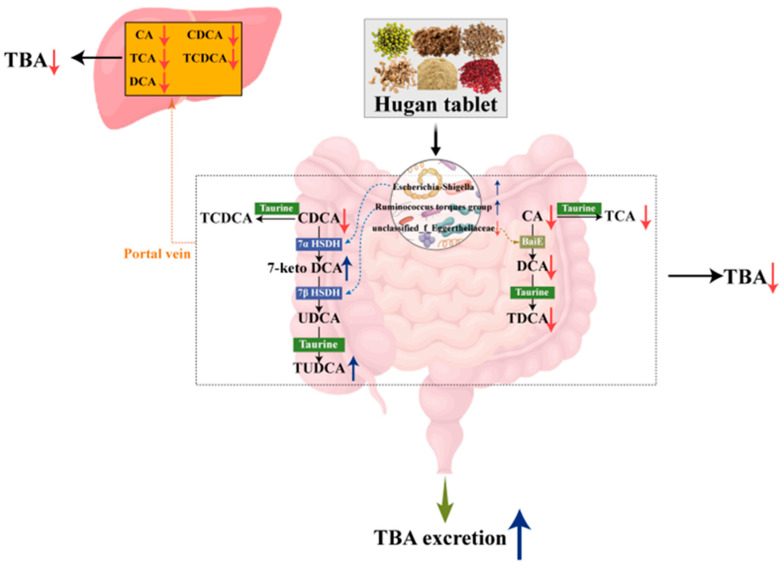
Schematic description of the mechanism of HGT in ameliorating NASH fibrosis.

HGT treatment increased the abundance of the *Ruminococcus_torques_group* and *Escherichia-Shigella*, which have potential in encoding 7αHSDH and 7βHSDH, while reducing the abundance of *unclassified_f_Eggerthellaceae*, which has the potential to express BaiE. Multiple intestinal ingredients of HGTs, including (R, S)-goitrin, schisandrin A, schisandrin C, schisandrin, schisantherin A, and schizandrol B, acted on 7αHSDH and 7βHSDH, facilitating the oxidation of CDCA to 7-keto LCA and its subsequent epimerization to UDCA, promoting BA excretion. Additionally, (R, S)-goitrin targeted BaiE, inhibiting the 7α-dehydroxylation of CA to DCA, thereby decreasing the abundance of intestinal DCA and TDCA as well as hepatic DCA, alleviating its pro-fibrotic effects (Figure 6). These combined effects contributed to the anti-fibrotic role of HGTs in NASH fibrosis mice. Our findings provide a scientific foundation for the clinical application of HGTs and offer novel perspectives on the active compounds of TCM formulations and their therapeutic targets. Large samples and clinical experiments are needed for subsequent verification of the active compounds and their potential mechanisms.

## Figures and Tables

**Table 1:** The analytic conditions of precursor ions of *m*/*z*, molecular formulas, and CEs of 31 BAs.

No.	Bile Acid		Molecular Formula	[M − H]^−^ (*m*/*z*)	NCE (eV)
	Abbreviation	Full Name			
1	3-oxoLCA	3-Oxolithocholic acid	C_24_H_38_O_3_	373.2737	−50
2	NorDCA	Nordeoxycholic acid	C_23_H_38_O_4_	377.2686	−22
3	12-ketoLCA	12-Ketolithocholic acid	C_24_H_38_O_4_	389.2686	−15
4	7-ketoLCA	7-Ketolithocholic acid	C_24_H_38_O_4_	389.2686	−15
5	12-oxoCDCA	12-Oxochenodeoxycholic acid	C_24_H_38_O_5_	405.2636	−15
6	7-keto DCA	7-Ketodeoxycholic acid	C_24_H_38_O_5_	405.2719	−15
7	alloLCA	Allolithocholic acid	C_24_H_40_O_3_	375.2894	−22
8	iso-alloLCA	Isoallolithocholic acid	C_24_H_40_O_3_	375.2894	−50
9	isoLCA	Isolithocholic acid	C_24_H_40_O_3_	375.2894	−50
10	CDCA	Chenodeoxycholic acid	C_24_H_40_O_4_	391.2843	−15
11	DCA	Deoxycholic acid	C_24_H_40_O_4_	391.2843	−15
12	HDCA	Hyodeoxycholic acid	C_24_H_40_O_4_	391.2843	−15
13	MDCA	Murideoxycholic acid	C_24_H_40_O_4_	391.2843	−15
14	UDCA	Ursodesoxycholic acid	C_24_H_40_O_4_	391.2843	−15
15	isoDCA	Isodeoxycholic acid	C_24_H_40_O_4_	391.2927	−15
16	alloCA	Allocholic acid	C_24_H_40_O_5_	407.2792	−10
17	CA	Cholic acid	C_24_H_40_O_5_	407.2792	−10
18	LCA	Lithocholic acid	C_24_H_40_O_3_	407.2792	−10
19	HCA	Hyocholic acid	C_24_H_40_O_5_	407.2792	−10
20	UCA	Ursocholic acid	C_24_H_40_O_5_	407.2792	−10
21	αMCA	alpha-Muricholic acid	C_24_H_40_O_5_	407.2792	−10
22	βMCA	beta-Muricholic acid	C_24_H_40_O_5_	407.2792	−10
23	TLCA	Taurolithocholic Acid	C_26_H_45_NO_5_S	482.2935	−80
24	TCDCA	Taurochenodeoxycholic acid	C_26_H_45_NO_6_S	498.2884	−72
25	TDCA	Taurodesoxycholic acid	C_26_H_45_NO_6_S	498.2884	−72
26	THDCA	Taurohyodeoxycholic acid	C_26_H_45_NO_6_S	498.2884	−72
27	TUDCA	Tauroursodeoxycholic acid	C_26_H_45_NO_6_S	498.2884	−72
28	TCA	Taurocholic acid	C_26_H_45_NO_7_S	514.2833	−80
29	THCA	Taurohyocholic acid	C_26_H_45_NO_7_S	514.2833	−80
30	TαMCA	Tauro-alpha-muricholic acid	C_26_H_45_NO_7_S	514.2833	−80
31	TβMCA	Tauro-beta-muricholic acid	C_26_H_45_NO_7_S	514.2833	−80

**Table 2:** HGTs reversed the differential BAs in the gut and liver.

Position	Class	BAs	VIP	Trend
Colonic contents	Primary BAs	CA	1.072	↓ *
Colonic contents	Primary BAs	CDCA	1.534	↓ **
Colonic contents	Primary BAs	TCA	1.954	↓ **
Colonic contents	Primary BAs	7-keto LCA	1.971	↑ **
Colonic contents	Primary BAs	TUDCA	1.473	↑ **
Colonic contents	Secondary BAs	DCA	1.982	↓ **
Colonic contents	Secondary BAs	TDCA	1.914	↓ **
Liver	Primary BAs	CA	1.550	↓ **
Liver	Primary BAs	CDCA	2.370	↓ **
Liver	Primary BAs	TCA	3.684	↓ **
Liver	Primary BAs	TCDCA	2.050	↓ *
Liver	Secondary BAs	DCA	1.299	↓ *

VIP: Variable importance in the projection for OPLS-DA, VIP ≥ 1. Trend: Downward arrow represents that HGTs reduced the abundance of BAs, upward arrow represents that HGTs increased the abundance of BAs, * *p* < 0.05, ** *p* < 0.01.

## Data Availability

The original contributions presented in the study are included in the article and Supplementary Materials. Further inquiries can be directed to the corresponding author.

## Supplementary Materials

The following supporting information can be downloaded at: https://www.mdpi.com/article/10.3390/metabo15070433/s1, Supplementary Figures: Supplementary Figure S1 (A) Core curves. (B) Pan curves. (C) Rank-abundance curves. (D) Chao index. (E) Simpson index. (F) Phylum level. (G) Family level. (H) Cladogram of Lefse analysis. (I) PICRUSt prediction analysis of gut microbiota between the Con group and the Mod group. (J) PICRUSt prediction analysis of gut microbiota between the Mod group and the HGT group; Supplementary Figure S2 (A) PCA plot of colonic contents. (B) Permutation testing of Con vs. Mod in colonic contents. (C) Permutation testing of Mod vs. HGTs in colonic contents. (D) Metabolic annotation of metabolites restored by HGTs (colonic contents). (E) PCA plot of the liver. (F) Permutation testing of Con vs. Mod in the liver. (G) Permutation testing of Mod vs. HGTs in the liver. (H) Metabolic annotation of metabolites restored by HGTs (liver); Supplementary Figure S3 (A) The concentration of DNA in colonic contents. (B) Number of OTUs. (C) Heatmap of log-adjusted relative abundance of intestinal bacterial phyla in colonic contents; Supplementary Figure S4 TBA levels in serum (A), feces (B), and liver (C). Overlapped chromatograms of transitions using PRM, (D) colonic contents, (E) liver. (F) Permutation testing in colonic contents. (G) Permutation testing in the liver; Supplementary Figure S5 Pearson’s correlation analysis. Supplementary Tables: Supplementary Table S1 Differential metabolites reversed by HGTs in the colonic contents; Supplementary Table S2 Differential metabolites reversed by HGTs in the liver; Supplementary Table S3 KEGG metabolic pathway enrichment (colonic contents); Supplementary Table S4 KEGG metabolic pathway enrichment (liver); Supplementary Table S5 Docking results of enzymes and ingredients of HGTs.
