# Supplementary material for: Integration of Pseudotargeted Metabolomics and Microbiomics Reveals That Hugan Tablets Ameliorate NASH with Liver Fibrosis in Mice by Modulating Bile Acid Metabolism via the Gut Microbiome
Source: Metabolites. 2025 Jun 24;15(7):433. doi: 10.3390/metabo15070433 (PMC12301009; doi:10.3390/metabo15070433)
Supplement: Supplementary file 1 [file metabolites-15-00433-s001.zip › metabolites-3601116-supplementary.pdf]

Integration of pseudotargeted metabolomics and microbiomics reveals Hugan Tablets ameliorate NASH with liver fibrosis in mice by modulating bile acid metabolism via gut microbiome

Supplementary Figures:

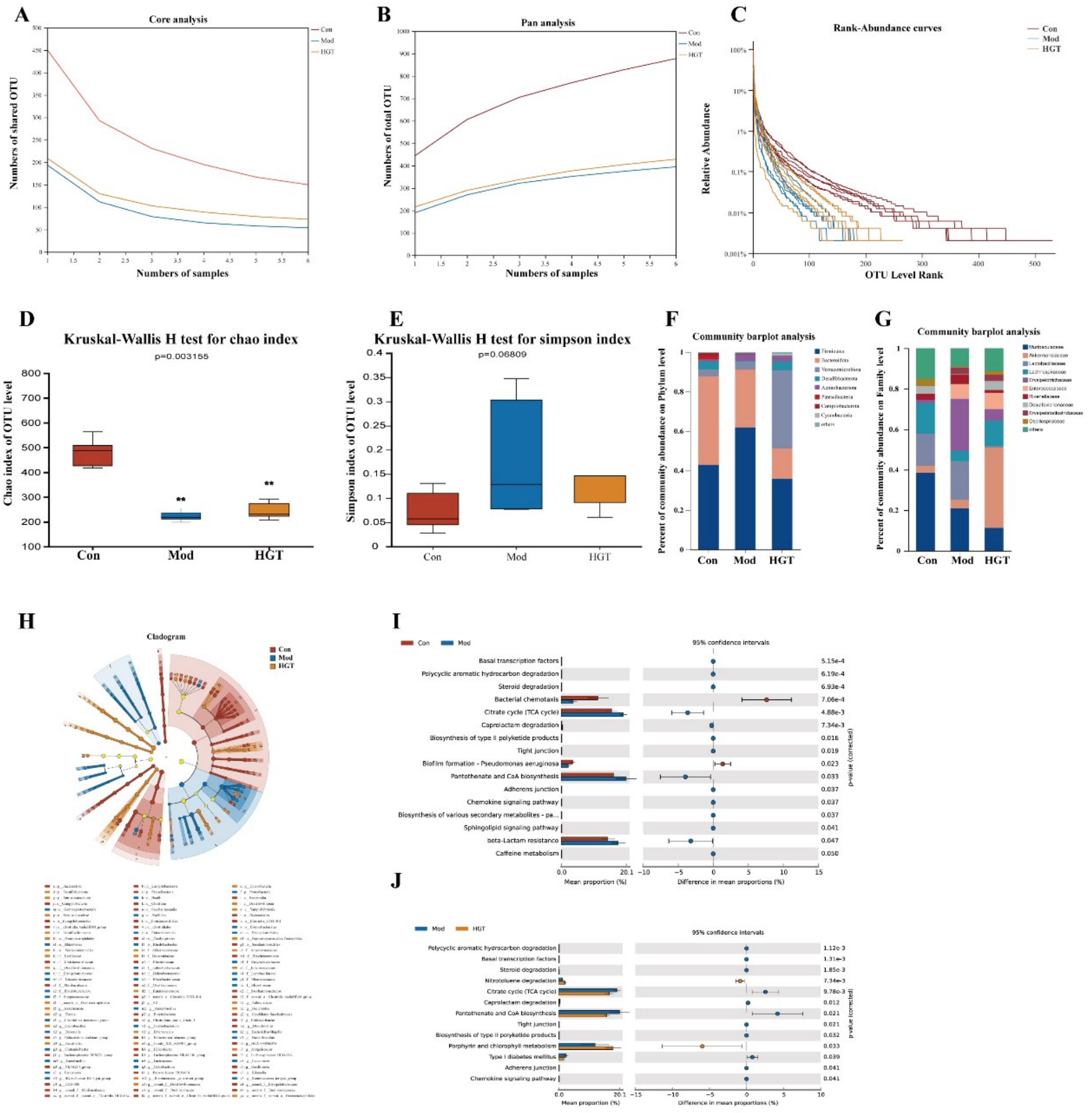

Figure S1 (**A**) Core curves; (**B**) Pan curves; (**C**) Rank-abundance curves; (**D**) Chao index; (**E**) Simpson index; (**F**) Phylum level; (**G**) Family level; (**H**) Cladogram of Lefse analysis; (**I**) PICRUSt prediction analysis of gut microbiota between Con group and Mod group; (**J**) PICRUSt prediction analysis of gut microbiota between Mod group and HGT group.

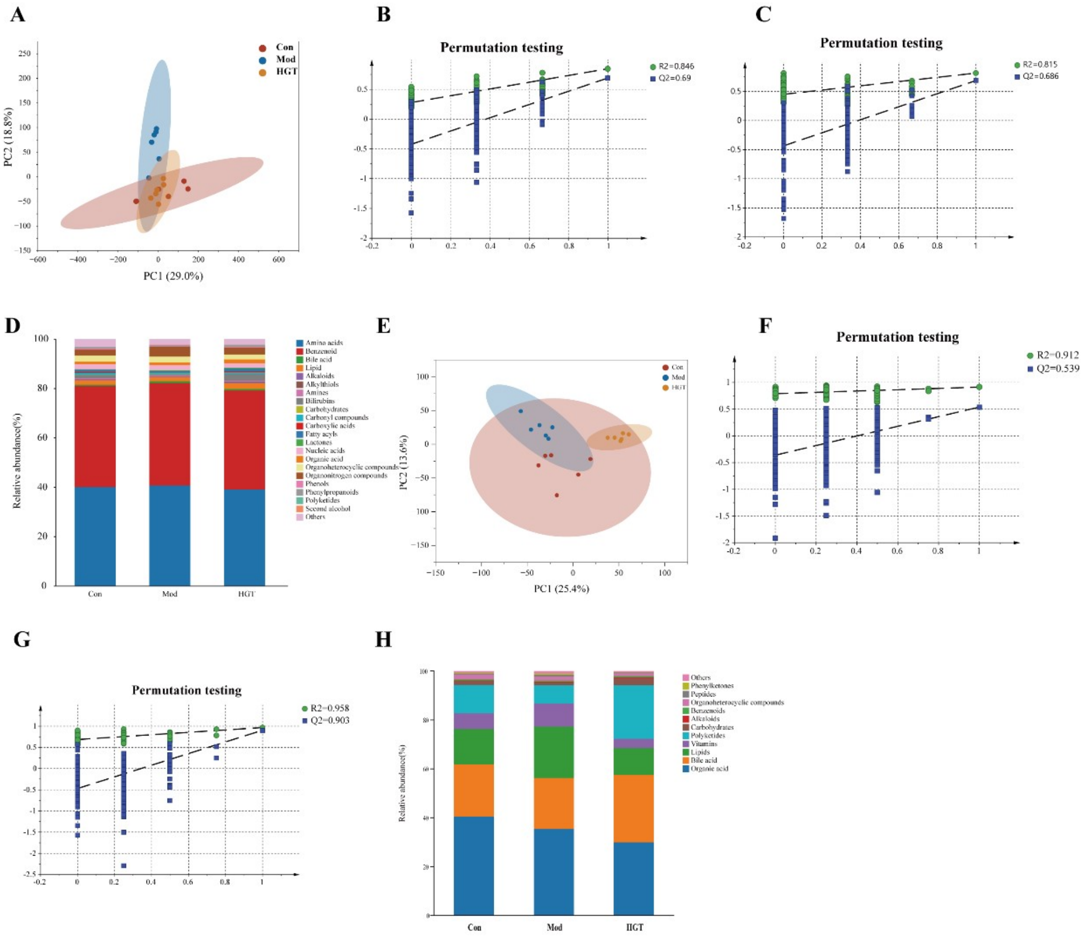

Figure S2. (**A**) PCA plot of colonic contents; (**B**) Permutation testing of Con vs. Mod in colonic contents; (**C**) Permutation testing of Mod vs. HGT in colonic contents; (**D**) Metabolic annotation of metabolites restored by HGT (colonic contents); (**E**) PCA plot of liver; (**F**) Permutation testing of Con vs. Mod in the liver; (**G**) Permutation testing of Mod vs. HGT in the liver; (**H**) Metabolic annotation of metabolites restored by HGT (liver).

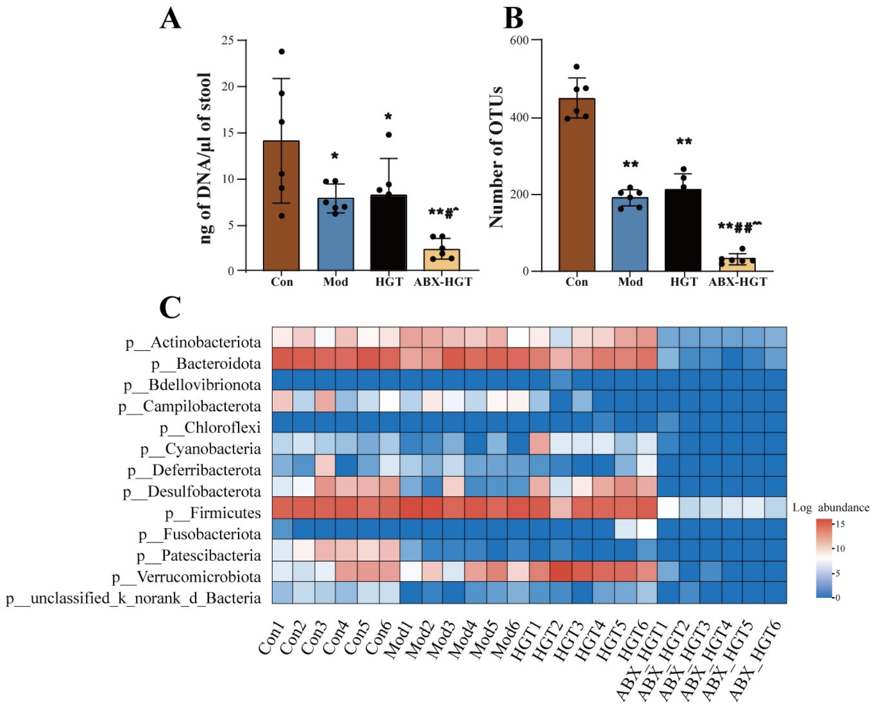

Figure S3. (**A**) The concentration of DNA in colonic contents. (**B**) Number of OTUs. (C) Heatmap of log-adjusted relative abundance of intestinal bacterial phylum in colonic contents.

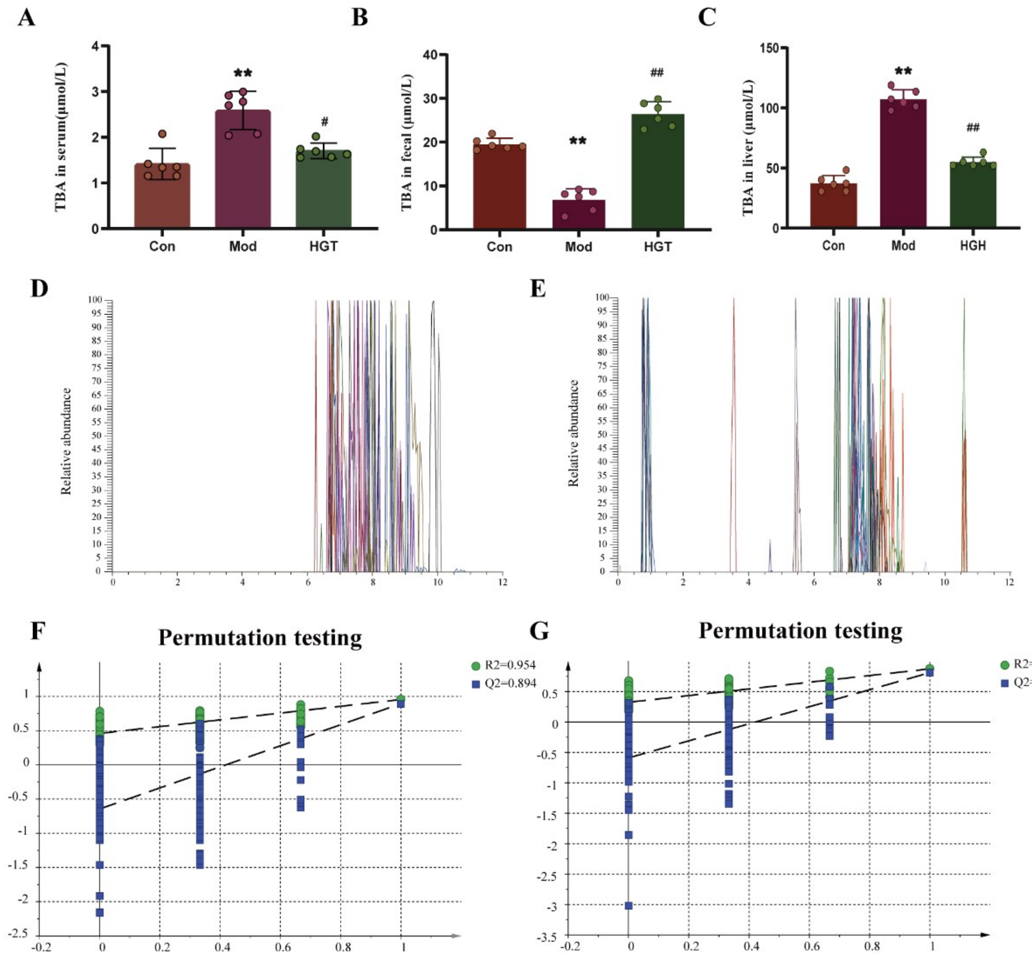

Figure S4. TBA levels in serum (**A**), fecal (**B**), and liver (**C**). Overlapped chromatograms of transitions using PRM, (**D**) colonic contents, (**E**) liver. (**F**) Permutation testing in colonic contents; (**G**) Permutation testing in the liver.

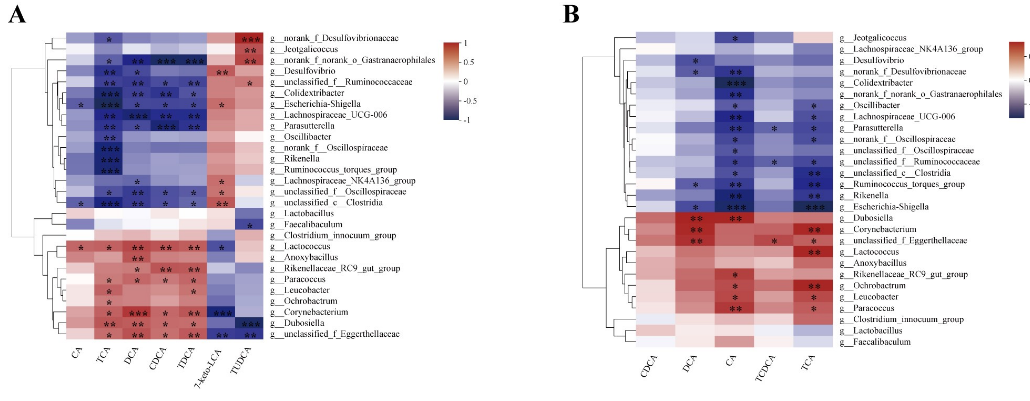

Figure S5. Pearson's correlation analysis. (**A**) Correlation of 28 potential key genera and differential BAs in colonic contents. (**B**) Correlation of 28 potential key genera and differential BAs in liver.

Supplementary Tables:

Table S1 Differential metabolites revered by HGT in the colonic contents

NO.	Metabolite	VIP	HMDB ID	Formula
1	Leucine	24.6515	HMDB0000687	$C_6H_{13}NO_2$
2	Choline	9.88505	HMDB0000097	$C_5H_{13}NO$
3	Urobilin	7.02756	HMDB0004160	$C_{33}H_{42}N_4O_6$
4	Methohexital	6.52603	HMDB0014617	$C_{14}H_{18}N_2O_3$
5	3-Amino-4-methylpentanoicacid	6.30156	HMDB0245808	$C_6H_{13}NO_2$
6	Mesobilirubinogen	6.23376	HMDB0001898	$C_{33}H_{44}N_4O_6$
7	3,3-Dimethyl-1,2-dithiolane	5.95084	HMDB0038974	$C_5H_{10}S_2$
8	D-Arginine	5.57329	HMDB0003416	$C_6H_{14}N_4O_2$
9	4-Nitroaniline	5.482	HMDB0246529	$C_6H_6N_2O_2$
10	Leucyl-Valine	5.20243	HMDB0028942	$C_{11}H_{22}N_2O_3$
11	D-Lacticacid	4.76174	HMDB0001311	$C_3H_6O_3$
12	Hydroxyzine	4.68577	HMDB0014697	$C_{21}H_{27}ClN_2O_2$
13	DL-Norvaline	4.63207	HMDB0251527	$C_5H_{11}NO_2$
14	3-beta-Hydroxy-4-beta-methyl-5-alpha-cholest-7-ene-4-alpha-carboxylate	4.43562	HMDB0011662	$C_{29}H_{48}O_3$
5	Alanylproline	4.09168	HMDB0028695	$C_8H_{14}N_2O_3$
16	D-Glutamicacid	4.08421	HMDB0003339	$C_5H_9NO_4$
17	MyristicanolA	3.94407	HMDB0041311	$C_{23}H_{30}O_8$
18	Lysine	3.92521	HMDB0000182	$C_6H_{14}N_2O_2$
19	O-Glutarylcarnitine	3.61681	HMDB0240779	$C_{12}H_{21}NO_6$
20	Spermidine	3.5198	HMDB000125	$C_7H_{19}N_3$

		8	7	
21	Guanosine	3.49407	HMDB0000133	$C_{10}H_{13}N_5O_5$
22	Thiosulfate	3.29544	HMDB0000257	$H_2O_3S_2$
23	7alpha-hydroxy-3-oxochol-4-en-24-oicAcid	3.20501	HMDB0062744	$C_{24}H_{36}O_4$
24	ProtoporphyrinIX	3.17781	HMDB0000241	$C_{34}H_{34}N_4O_4$
25	Guanine	3.17275	HMDB0000132	$C_5H_5N_5O$
26	Valylvaline	3.15951	HMDB0029140	$C_{10}H_{20}N_2O_3$
27	Arginylproline	2.99802	HMDB0028717	$C_{11}H_{21}N_5O_3$
28	N-[(3s)-2-Oxotetrahydrofuran-3-Yl] butanamide	2.9012	HMDB0247190	$C_8H_{13}NO_3$
29	4alpha-Formyl-4beta-methyl-5alpha-cholesta-8,24-dien-3beta-ol	2.8964	HMDB0012167	$C_{29}H_{46}O_2$
30	Dethiobiotin	2.84372	HMDB0003581	$C_{10}H_{18}N_2O_3$
31	D-Proline	2.75786	HMDB0003411	$C_5H_9NO_2$
32	Inosine	2.70023	HMDB0000195	$C_{10}H_{12}N_4O_5$
33	Piperidine	2.65441	HMDB0034301	$C_5H_{11}N$
34	D-Asparticacid	2.58875	HMDB0006483	$C_4H_7NO_4$
35	3-Methylsulfolene	2.55544	HMDB0059667	$C_5H_8O_2S$
36	Alanylproline	2.52099	HMDB0028695	$C_8H_{14}N_2O_3$
37	Pseudouridine	2.51832	HMDB0000767	$C_9H_{12}N_2O_6$
38	2-Hydroxyphenethylamine	2.5134	HMDB0001065	$C_8H_{11}NO$
39	delta-Valerolactone	2.50305	HMDB0250981	$C_5H_8O_2$
40	4-Hydroxybenzaldehyde	2.48435	HMDB0011718	$C_7H_6O_2$
41	Ecgonine	2.48276	HMDB0006548	$C_9H_{15}NO_3$

42	Lysylproline	2.41698	HMDB0028959	$C_{11}H_{21}N_3O_3$
43	D-threo-Isocitricacid	2.38904	HMDB0001874	$C_6H_8O_7$
44	Eugenol	2.388	HMDB0005809	$C_{10}H_{12}O_2$
45	L-Theanine	2.36499	HMDB0034365	$C_7H_{14}N_2O_3$
46	EriojaposideB	2.32334	HMDB0038029	$C_{25}H_{40}O_{11}$
47	Asparaginyl-Proline	2.30931	HMDB0028739	$C_9H_{15}N_3O_4$
48	S-(2-Methylpropionyl)-dihydrolipoamide-E	2.30741	HMDB0006868	$C_{12}H_{23}NO_2S_2$
49	Thymidine	2.25956	HMDB0000273	$C_{10}H_{14}N_2O_5$
50	Uracil	2.23629	HMDB0000300	$C_4H_4N_2O_2$
51	2-C-methyl-D-erythritol-4-phosphate	2.19278	HMDB0304061	$C_5H_{13}O_7P$
52	D-Pipecolicacid	2.18831	HMDB0005960	$C_6H_{11}NO_2$
53	Sulfate	2.18022	HMDB0001448	H_2O_4S
54	Lysylleucine	2.17483	HMDB0028955	$C_{12}H_{25}N_3O_3$
55	Fumaricacid	2.17085	HMDB0000134	$C_4H_4O_4$
56	N-[(3a,5b,7a)-3-hydroxy-24-oxo-7-(sulfooxy) cholan-24-yl]-Glycine	2.13981	HMDB0002496	$C_{26}H_{43}NO_8S$
57	L-Lysopine	2.03713	HMDB0033675	$C_9H_{18}N_2O_4$
58	gamma-Glutamylvaline	1.96628	HMDB0011172	$C_{10}H_{18}N_2O_5$
59	2'-Deoxymugineicacid	1.94063	HMDB0033909	$C_{12}H_{20}N_2O_7$
60	gamma-Glutamylleucine	1.90009	HMDB0011171	$C_{11}H_{20}N_2O_5$
61	cis-Aconiticacid	1.89266	HMDB0000072	$C_6H_6O_6$
62	Salsolinol	1.89188	HMDB0042012	$C_{10}H_{13}NO_2$
63	dihydro-3-hydroxy-4,4-dimeth	1.8802	HMDB030390	$C_6H_{10}O_3$

	yl-2(3H)-Furanone	8	2	
64	Taurocholicacid	1.86797	HMDB0000036	$C_{26}H_{45}NO_7S$
65	Ureidoisobutyricacid	1.84854	HMDB0002031	$C_5H_{10}N_2O_3$
66	2-Isopropyl-3-oxosuccinate	1.81895	HMDB0012149	$C_7H_{10}O_5$
67	3-Isopropylmalicacid	1.79562	HMDB0012156	$C_7H_{12}O_5$
68	Leucyl-Arginine	1.76297	HMDB0028923	$C_{12}H_{25}N_5O_3$
69	Convicine	1.76079	HMDB0250445	$C_{10}H_{15}N_3O_8$
70	Heptane-1-thiol	1.74796	HMDB0032304	$C_7H_{16}S$
71	L-2-Amino-3-oxobutanoicacid	1.73328	HMDB0006454	$C_4H_7NO_3$
72	gamma-Glutamyl-S-methylcysteinyl-beta-alanine	1.72183	HMDB0039504	$C_{12}H_{21}N_3O_6S$
73	Maltol	1.6799	HMDB0030776	$C_6H_6O_3$
74	GomisinA	1.67572	HMDB0252910	$C_{23}H_{28}O_7$
75	Leucylproline	1.66734	HMDB0011175	$C_{11}H_{20}N_2O_3$
76	Allopurinol	1.6501	HMDB0014581	$C_5H_4N_4O$
77	1-Hydroxyisoquinoline	1.63489	HMDB0243900	C_9H_7NO
78	Acamprosate	1.63294	HMDB0014797	$C_5H_{11}NO_4S$
79	Marimastat	1.62908	HMDB0014924	$C_{15}H_{29}N_3O_5$
80	Desglucocheirotoxin	1.59884	HMDB0034362	$C_{29}H_{42}O_{10}$
81	D-2-Hydroxyglutaricacid	1.58778	HMDB0000606	$C_5H_8O_5$
82	Benzaldehyde	1.57305	HMDB0006115	C_7H_6O
83	Palmitoylethanolamide	1.56712	HMDB0002100	$C_{18}H_{37}NO_2$
84	keratansulfateI	1.56656	HMDB0062483	$C_{15}H_{22}N_2O_3$
85	N-Acetyl-L-glutamate5-semia	1.5609	HMDB000648	$C_7H_{11}NO_4$

	ldehyde	4	8	
86	L-Threose	1.55769	HMDB0002649	$C_4H_8O_4$
87	Prolyl-Glutamine	1.53	HMDB0029015	$C_{10}H_{17}N_3O_4$
88	Aspartame	1.52432	HMDB0001894	$C_{14}H_{18}N_2O_5$
89	Hyodeoxycholicacid	1.52369	HMDB0000733	$C_{24}H_{40}O_4$
90	Leucyl-Asparagine	1.51378	HMDB0028924	$C_{10}H_{19}N_3O_4$
91	Propenoylcarnitine	1.49555	HMDB0013124	$C_{10}H_{17}NO_4$
92	Bilirubin	1.49389	HMDB0000054	$C_{33}H_{36}N_4O_6$
93	3-Hydroxypicolinicacid	1.45438	HMDB0013188	$C_6H_5NO_3$
94	Taurochenodesoxycholicacid	1.45344	HMDB0000951	$C_{26}H_{45}NO_6S$
95	D-Alanyl-D-alanine	1.44931	HMDB0003459	$C_6H_{12}N_2O_3$
96	Xanthine	1.43158	HMDB0000292	$C_5H_4N_4O_2$
97	Deoxyguanosine	1.41485	HMDB0000085	$C_{10}H_{13}N_5O_4$
98	N-(1-Deoxy-1-fructosyl) leucine	1.37624	HMDB0037840	$C_{12}H_{23}NO_7$
99	Glutamylglycine	1.37354	HMDB0028819	$C_7H_{12}N_2O_5$
100	4,4-Dimethylcholesta-8,14,24-trienol	1.36985	HMDB0001023	$C_{29}H_{46}O$
101	D-Phenylalanine	1.35587	HMDB0250791	$C_9H_{11}NO_2$
102	Deoxyuridine	1.35432	HMDB0000012	$C_9H_{12}N_2O_5$
103	2-Ethyl-4,5-dimethyloxazole	1.34415	HMDB0037865	$C_7H_{11}NO$
104	TuliposideB	1.33548	HMDB0303141	$C_{11}H_{18}O_9$
105	Stearidonicacid	1.31711	HMDB0006547	$C_{18}H_{28}O_2$
106	N6-Acetyl-L-lysine	1.28043	HMDB0000206	$C_8H_{16}N_2O_3$
107	D-Malicacid	1.2799	HMDB003151	$C_4H_6O_5$

		7	8	
108	Indole	1.27384	HMDB0000738	C_8H_7N
109	1,5-Naphthalenediamine	1.27083	HMDB0244231	$C_{10}H_{10}N_2$
110	Dihydrocortisol	1.26526	HMDB0003259	$C_{21}H_{32}O_5$
111	Sulfoaceticacid	1.26422	HMDB0258590	$C_2H_4O_5S$
112	Valyl-Arginine	1.25711	HMDB0304792	$C_{11}H_{23}N_5O_3$
113	Alanylleucine	1.247	HMDB0028691	$C_9H_{18}N_2O_3$
114	Methionine	1.22797	HMDB0000696	$C_5H_{11}NO_2S$
115	4-Ethoxybenzaldehyde	1.22584	HMDB0033970	$C_9H_{10}O_2$
116	Diaminopimelicacid	1.2244	HMDB0001370	$C_7H_{14}N_2O_4$
117	Chorismate	1.21813	HMDB0012199	$C_{10}H_{10}O_6$
118	(3S)-3,6-Diaminohexanoate	1.19013	HMDB0012114	$C_6H_{14}N_2O_2$
119	Deoxyinosine	1.16985	HMDB0000071	$C_{10}H_{12}N_4O_4$
120	Deoxycholicacid	1.16166	HMDB0000626	$C_{24}H_{40}O_4$
121	Vanylglycol	1.15364	HMDB0001490	$C_9H_{12}O_4$
122	3-Oxo-5beta-cholanoicacid	1.14779	HMDB0304950	$C_{24}H_{38}O_3$
123	3-Methylcrotonylglycine	1.13472	HMDB0000459	$C_7H_{11}NO_3$
124	Leucyl-Glutamine	1.11915	HMDB0028927	$C_{11}H_{21}N_3O_4$
125	2-Hydroxycaproicacid	1.11241	HMDB0001624	$C_6H_{12}O_3$
126	Terbutaline	1.11184	HMDB0015009	$C_{12}H_{19}NO_3$
127	Lysyl-Phenylalanine	1.1073	HMDB0304786	$C_{15}H_{23}N_3O_3$
128	Chenodeoxyglycocholicacid	1.1046	HMDB0006898	$C_{26}H_{43}NO_5$
129	Trimetaphosphoricacid	1.1013	HMDB005992	$H_3O_9P_3$

		9	1	
130	2,4-Toluenediamine	1.0976	HMDB0041799	$C_7H_{10}N_2$
131	Prolyl-Methionine	1.09592	HMDB0029023	$C_{10}H_{18}N_2O_3S$
132	Oleamide	1.06831	HMDB0002117	$C_{18}H_{35}NO$
133	Coumarin	1.059	HMDB0001218	$C_9H_6O_2$
134	Levetiracetam	1.04798	HMDB0015333	$C_8H_{14}N_2O_2$
135	1-Aminocyclopropanecarboxylicacid	1.0445	HMDB0036458	$C_4H_7NO_2$
136	(Z)- [(4-hydroxyphenyl) acetaldehydeoxime]	1.03948	HMDB0304004	$C_8H_9NO_2$
137	1-Hexanethiol	1.03129	HMDB0039806	$C_6H_{14}S$
138	Beta-Tyrosine	1.02265	HMDB0003831	$C_9H_{11}NO_3$
139	beta-D-ribosylnicotinate	1.0195	HMDB0304540	$C_{11}H_{13}NO_6$
140	(S)-5-Amino-3-oxohexanoate	1.01572	HMDB0012131	$C_6H_{11}NO_3$
141	Menoctone	1.01504	HMDB0254439	$C_{24}H_{32}O_3$
142	Phenylacetaldehyde	1.01349	HMDB0006236	C_8H_8O
143	2'-Aminoacetophenone	1.00992	HMDB0032630	C_8H_9NO
144	6-Hydroxypseudooxynicotine	1.00239	HMDB0240264	$C_{10}H_{14}N_2O_2$
145	1-Pyrroline-4-hydroxy-2-carboxylate	1.00214	HMDB0002234	$C_5H_7NO_3$
146	Asparaginyl-Valine	1.00133	HMDB0028744	$C_9H_{17}N_3O_4$
147	2-Furoicacid	1.00107	HMDB0000617	$C_5H_4O_3$

Table S2 Differential metabolites reversed by HGT in the liver

NO.	Metabolite	VIP	HMDB ID
1	Lactic acid	19.9584	HMDB0000190
2	Maltol	13.8309	HMDB0030776
3	LysoPC (18:2/0:0)	6.80736	HMDB0010386

4	Niacinamide	5.66658	HMDB0001406
5	1D-chiro-inositol	4.75084	HMDB0240209
6	Taurine	4.66107	HMDB0000251
7	1-Palmitoylglycerophosphocholine	4.60966	HMDB0256091
8	(S)-Methylmalonate semialdehyde	4.55521	HMDB0002217
9	LysoPC (20:4/0:0)	3.90635	HMDB0010395
10	LysoPC (22:6(4Z,7Z,10Z,13Z,16Z,19Z)/0:0)	3.78774	HMDB0010404
11	Cholesterol sulfate	3.08082	HMDB0000653
12	Pantolactone	2.88705	HMDB0059876
13	LysoPC (18:1/0:0)	2.72946	HMDB0002815
14	Sedoheptulose	2.58937	HMDB0003219
15	Phosphate	2.56646	HMDB0001429
16	D-Proline	2.47669	HMDB0003411
17	LysoPI(20:4(5Z,8Z,11Z,14Z)/0:0)	2.37783	HMDB0061690
18	Dihydrolipoate	2.08527	HMDB0012210
19	1,5-Anhydro-d-mannitol	2.07606	HMDB0244228
20	5-Acetylamino-6-formylamino-3-methyluracil	1.9996	HMDB0011105
21	3-Methyloxindole	1.88694	HMDB0304943
22	Symmetric dimethylarginine	1.8262	HMDB0003334
23	O-Glutarylcarnitine	1.82619	HMDB0240779
24	LysoPE (22:6(4Z,7Z,10Z,13Z,16Z,19Z)/0:0)	1.70464	HMDB0011526
25	Platelet-activating factor	1.68566	HMDB0062195
26	4-Undecylbenzenesulfonic acid	1.6021	HMDB0032549
27	D-threo-Isocitric acid	1.60115	HMDB0001874
28	Taurochenodeoxycholic acid	1.58038	HMDB0000951
29	D-Erythrose	1.50848	HMDB0250746
30	3- [(2Z)-1-Oxo-2-buten-2-yl] pentanedioic acid	1.42488	HMDB0033091
31	Kynurenine	1.37644	HMDB0000684
32	6-Methylnicotinamide	1.20175	HMDB0013704
33	Glycerophosphocholine	1.19021	HMDB0000086
34	20R-Camptothecin	1.18795	HMDB0245602
35	Phenylethylmalonamide	1.0967	HMDB0245113
36	1-(4-Hydroxyphenyl)-2-aminoethanol	1.02222	HMDB0004825
37	Chenodeoxyglycocholate	1.01519	HMDB0006898

Table S3. KEGG metabolic pathway enrichment (Colonic contents)

Pathway Name	Match Status	*p*	-log(*p*)	Impact
D-Amino acid metabolism	5\15	1.2018E-5	4.9202	1
Purine metabolism	7\71	8.2605E-4	3.083	0.05525
Sulfur metabolism	2\8	0.013109	1.8824	0.21277
Pyrimidine metabolism	3\39	0.057387	1.2412	0.14895
Citrate cycle (TCA cycle)	2\20	0.074931	1.1253	0.07984

beta-Alanine metabolism	2\21	0.081654	1.088	0
Primary bile acid biosynthesis	3\46	0.085544	1.0678	0.06855
Pyruvate metabolism	2\23	0.095634	1.0194	0.07682
Alanine, aspartate and glutamate metabolism	2\28	0.13319	0.87552	0.0024
Porphyrin metabolism	2\31	0.15713	0.80374	0.14286
Valine, leucine and isoleucine biosynthesis	1\8	0.16967	0.7704	0
Taurine and hypotaurine metabolism	1\8	0.16967	0.7704	0
Glycine, serine and threonine metabolism	2\34	0.18184	0.74032	0
Arginine and proline metabolism	2\36	0.19863	0.70195	0.06628
Biotin metabolism	1\10	0.20751	0.68297	0
Phenylalanine metabolism	1\10	0.20751	0.68297	0.14286
Tyrosine metabolism	2\42	0.25	0.60207	0.02463
alpha-Linolenic acid metabolism	1\13	0.26114	0.58313	0
Arginine biosynthesis	1\14	0.27822	0.55562	0
Pantothenate and CoA biosynthesis	1\20	0.37293	0.42837	0
Glutathione metabolism	1\28	0.48063	0.31819	0.00719
Lysine degradation	1\30	0.5046	0.29705	0
Glyoxylate and dicarboxylate metabolism	1\32	0.52751	0.27777	0.02381
Cysteine and methionine metabolism	1\33	0.53857	0.26876	0.10446
Glycerophospholipid metabolism	1\36	0.57026	0.24392	0.02582
Valine, leucine and isoleucine degradation	1\40	0.60925	0.2152	0
Steroid biosynthesis	1\41	0.61845	0.20869	0.06644
Steroid hormone biosynthesis	1\79	0.84754	0.07184	0

Table S4. KEGG metabolic pathway enrichment (Liver)

Pathway Name	Match Status	*p*	-log(*p*)	Impact
Taurine and hypotaurine metabolism	2/8	0.0018211	2.7397	0.42857
Primary bile acid biosynthesis	3/46	0.0059243	2.2274	0.06809
Glycerophospholipid metabolism	2/36	0.035813	1.446	0.0655
Caffeine metabolism	1/12	0.097847	1.0095	0
D-Amino acid metabolism	1/15	0.12089	0.91762	0
Nicotinate and nicotinamide metabolism	1/15	0.12089	0.91762	0.1943
Ether lipid metabolism	1/20	0.15808	0.80111	0

Propanoate metabolism	1/22	0.17255	0.76308	0
Pentose phosphate pathway	1/23	0.1797	0.74545	0
Pyruvate metabolism	1/23	0.1797	0.74545	0
Glycolysis / Gluconeogenesis	1/26	0.2008	0.69723	0
Arginine and proline metabolism	1/36	0.26757	0.57255	0
Valine, leucine and isoleucine degradation	1/40	0.29281	0.53341	0.0301
Tryptophan metabolism	1/41	0.29899	0.52434	0.09417
Steroid hormone biosynthesis	1/79	0.50012	0.30093	0

Table S5 Docking results of enzymes and ingredients of HGT

BAs-metabolizing enzymes	Ingredients	Libdock score	Binding energy (kcal/mol)
7αHSDH	(R, S)-goitrin	58.6104	-1.6482
	Schisandrin	80.2	-10.6022
	Schisandrin A	87.7353	-6.4354
	Schisandrin C	116.017	-2.9595
	Schisantherin A	119.191	-12.2798
	Schizandrol B	101.467	-7.1279
7βHSDH	(R, S)-goitrin	55.4021	-6.5271
	Schisandrin	88.8492	-5.1213
BaiE	(R, S)-goitrin	48.4252	-7.0607
